# Critical Thinking and Clinical Decision Making Among Registered Nurses in Clinical Practice: A Systematic Review and Meta-Analysis

**DOI:** 10.3390/nursrep15050175

**Published:** 2025-05-20

**Authors:** Nur Hidayah Zainal, Md Asiful Islam, Nur Syahmina Rasudin, Zakira Mamat, Tengku Muhammad Hanis, Wan Shakira Rodzlan Hasani, Kamarul Imran Musa

**Affiliations:** 1 Department of Community Medicine, School of Medical Sciences, Universiti Sains Malaysia, Kubang Kerian 16150, Kelantan, Malaysia; 2 Department of Biomedical Science and Physiology, School of Pharmacy and Life Sciences, Faculty of Science and Engineering, University of Wolverhampton, Wolverhampton WV1 1LY, UK; 3 Biomedicine Programme, School of Health Sciences, Universiti Sains Malaysia, Kubang Kerian 16150, Kelantan, Malaysia; 4 Nursing Programme, School of Health Sciences, Universiti Sains Malaysia, Kubang Kerian 16150, Kelantan, Malaysia; 5 Department of Community Medicine, Faculty of Medicine, Universiti Sultan Zainal Abidin, Medical Campus, Jalan Sultan Mahmud, Kuala Terengganu 20400, Terengganu, Malaysia; 6 Nursing Research Unit, Nursing Division, Ministry of Health Malaysia, Level 3, Block E7, Parcel E, Persint 1, Federal Government Administrative Centre, Putrajaya 62590, Malaysia

**Keywords:** critical thinking, clinical decision making, nurses, nursing, clinical practice, systematic review, meta-analysis

## Abstract

**Background:** Critical thinking is fundamental for registered nurses (RNs) when making clinical decisions, which impact patient outcomes. This review aimed to identify studies on critical thinking and clinical decision making among nurses in clinical practice and synthesize their findings based on the regional area, observed findings, and predictive factors, and to assess the measurement tools used. **Methods**: A comprehensive search of the PubMed, Web of Science, CINAHL, and SCOPUS databases up to December 2024 was conducted in accordance with the PRISMA guidelines. The Newcastle–Ottawa Scale was used to assess the quality of included studies. Studies with similarly themed components were grouped for narrative synthesis. A meta-analysis of random-effects model calculations was performed. **Results**: This review included forty studies (twenty-four on CT, twelve on CDM, four on both) from various WHO regions, revealing diverse findings on observed skills. Ten CT and four CDM measurement tools were identified. Many studies also explored individual and group-level predictive factors for these skills. Meta-analyses of four common tools (CCTDI, NCT4P, CDMNS, and NDMI) showed significant heterogeneity, with statistically significant pooled mean scores. **Conclusions**: The synthesis highlights the global research on nurses’ critical thinking and clinical decision making, including the exploration of various predictive factors. However, the significant heterogeneity in the findings from meta-analyses of commonly used measurement tools underscores a need for more standardized measurement and analytical approaches, such as multilevel modeling, to better account for the hierarchical nature of potential predictive factors (individual and group levels), which would allow for more reliable comparisons and stronger conclusions in this field.

## 1. Introduction

In the dynamic landscape of healthcare, the ability of nurses to engage in critical thinking (CT) and clinical decision making (CDM) plays a pivotal role in ensuring the delivery of safe, effective, and patient-centered care [1,2]. With patients’ lives and well-being at stake, nurses are constantly faced with complex and rapidly evolving clinical situations that demand quick and accurate decision making. Critical thinking, defined as the deliberate and systematic thought process used to guide one’s actions, is a fundamental skill that is crucial for clinical decision making among nurses [3,4,5].

**Figure 1:**
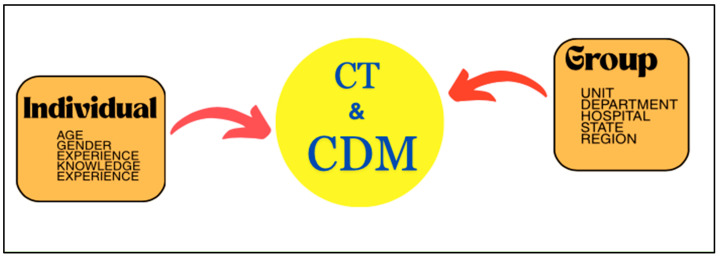
This is a basic conceptual model of factors influencing critical thinking (CT) and clinical decision making (CDM) in registered nurses.

Over the years, healthcare systems worldwide have undergone significant transformations, necessitating a re-evaluation of the role of registered nurses (RNs) in clinical practice. The increasing acuity of patients, advances in medical technology, and the ever-expanding body of medical knowledge have placed new demands on nursing professionals. Consequently, it is essential to investigate how RNs engage in CT and CDM in their clinical practice [6,7]. Critical thinking in nursing clinical practice refers to the ability of nurses to think logically, make reasoned judgments, and solve complex problems in the context of patient care [8,9]. It involves the use of cognitive skills and intellectual abilities to analyze information, assess situations, and make informed decisions for the benefit of the patient’s health and well-being. Meanwhile, clinical decision making in nursing clinical practice refers to the ability of nurses to systematically assess and analyze patient information, consider relevant evidence and factors, and make well-informed and patient-centered decisions about the care and treatment of their patients [10,11]. Both skills are crucial for ensuring safe and effective nursing care. Figure 1 shows a basic model illustrating that the levels of critical thinking (CT) and clinical decision-making (CDM) skills in registered nurses are influenced by both individual characteristics (e.g., age, gender, experience, and knowledge) and group/contextual factors (e.g., unit, department, hospital, state, and region). The evaluation of CT and CDM in nursing clinical practice can vary depending on the healthcare institution, educational program, or regulatory body. Some commonly used and well-regarded methods for evaluation include direct observation, case studies, simulated patient scenarios, self-assessment, and reflection.

A systematic review follows a rigorous methodology, employing transparent and replicable search strategies to identify relevant studies [12,13]. While several reviews have explored CT and CDM skills individually among nurses, none has comprehensively examined both together. Previous reviews, often focused on nursing education, might have shown a positive correlation between critical thinking and clinical decision-making skills in nursing students [14,15,16,17]. This suggests that education plays a role, but our review specifically focuses on practicing RNs. Furthermore, our limited search revealed no recent reviews synthesizing findings on both CT and CDM skills together, along with their predictive factors at both the individual and group levels, specifically within the population of practicing registered nurses. Therefore, this systematic review and meta-analysis endeavor to bridge this critical lacuna in the literature by scrupulously scrutinizing the collective evidence pertaining to CT and CDM among RNs in clinical practice. This scrutiny probes a fundamental inquiry: What are the levels of CT and CDM among RNs, and what factors, at both the individual and group levels, predict proficiency in these essential skills? Consequently, we aspire that this review, with its meta-analysis of available measurement tools, will furnish invaluable insights into the current state of CT and CDM in nursing practice, informing the development of targeted interventions to enhance these crucial competencies. This review is of paramount importance to nurse educators, administrators, policymakers, and clinicians alike. It can inform the development of educational programs, training initiatives, and clinical guidelines that enhance nurses’ CT skills and facilitate more effective CDM. Ultimately, the goal is to improve patient outcomes and the quality of care provided by RNs, ensuring they are well prepared to meet the evolving challenges of contemporary healthcare settings.

## 2. Methods

### 2.1. Protocol and Registration

We followed the revised Preferred Reporting Items for Systematic Reviews and Meta-Analyses (PRISMA) 2020 statement for this review [18] (S1 Checklist). The protocol for this review was registered in the International Prospective Register of Systematic Reviews (PROSPERO) (CRD42023447842).

### 2.2. Search Strategy

We searched the electronic databases PubMed, Scopus, CINAHL, and Web of Science (WoS) to identify eligible studies. We cross-checked all eligible articles from the reference lists of included articles. We also searched Google Scholar to identify articles not indexed in the major electronic databases. All databases were searched from their inception through to December 2024. Our search strategy included terms for “critical thinking”, “clinical decision-making”, “clinical reasoning”, “clinical judgment”, “nurse”, “nurses”, and “nursing”. The search was limited to studies published in English. The detailed search terms for each database are presented in Supplementary Table S1.

### 2.3. Study Selection

The Mendeley Reference Management software (https://www.mendeley.com accessed on 25 December 2024) was used to store, organize, and manage all references. Prior to screening, all search results were imported into Mendeley, and duplicate papers were removed by one author (N.H.Z.). The screening process was divided into two phases. In the first phase, four authors (N.H.Z., M.A.I., T.M.H., and W.S.R.H.) independently screened titles and abstracts to identify potentially eligible studies and exclude obviously irrelevant ones. Studies were included if they (1) involved registered nurses (RNs) actively working in clinical practice; (2) assessed or measured either critical thinking (CT) skills, clinical decision-making (CDM) skills, or both, using any validated or described measurement tool; (3) employed an observational study design such as cross-sectional, and reported on the assessment of CT and/or CDM skills; and (4) were published in the English language. Reviews, meta-analyses, letters to the editor, editorials, commentaries, conference abstracts, and gray literature such as unpublished reports and theses that did not undergo peer review were excluded.

We retrieved the full-text articles for potentially relevant studies in the second phase of screening. Three review authors (N.H.Z., T.M.H., and W.S.R.H.) independently screened these full-text articles. The authors reviewed the studies based on predetermined eligibility criteria. The eligibility criteria for this phase were as follows: (a) studies reporting perceptions of CT and/or CDM in RNs; (b) studies reporting predictive factors of CT and/or CDM; (c) studies using measurement tools to evaluate CT and/or CDM; (d) studies primarily targeting registered/clinical nurses working shifts; and (e) quantitative research. Due to differences in definitions, characteristics, and focus on various aspects of nursing care, clinical reasoning and clinical judgment were excluded. The reasons for inclusion or exclusion of each study were recorded. Disagreements during the screening process were resolved through discussion, or, when necessary, consultation with the fifth and sixth review authors (N.S.R. and Z.M.). If consensus could not be reached, another author (K.I.M.) acted as an arbiter. The selection process was recorded, and a PRISMA flow diagram was completed [18].

### 2.4. Data Extraction and Management

In systematic reviews and meta-analyses, tabulation and grouping techniques are essential methods for summarizing and organizing data extracted from individual studies. These techniques help researchers present and analyze the findings of included studies in a structured and comprehensible manner. We created tables to record key information from each included study and used narrative synthesis to summarize and interpret findings when meta-analysis was not possible or appropriate. In narrative synthesis, researchers group studies thematically and provide a descriptive synthesis of the evidence. Three reviewer authors (N.H.Z., T.M.H., and W.S.R.H.) independently extracted the data according to guidance from the Cochrane Handbook for Systematic Reviews of Interventions. We used a standardized data extraction form created using Microsoft Excel (Table S2) for study characteristics and outcome data. One reviewer (N.H.Z.) conducted a full abstraction of all data, and two other reviewers (T.M.H. and W.S.R.H.) verified its accuracy. The authors then undertook a thematic synthesis of the collated results. Themes were pre-specified a priori based on categories from previous research but were adapted based on the information reported in the included studies to analyze the results and thematize important, similar data and patterns [13,16]. The papers were read through several times to ensure that all crucial data impacting nursing practice were integrated. From all eligible articles, we abstracted the first author’s name, year of publication, country, study design, study population, aim of study, skills assessed, skill scores, skill levels, predictive factors assessed, and the tools used. We contacted the authors to obtain details if no score or level was reported.

The main outcomes in this review were the level of CT and/or the level of CDM, the predictive factors, and an assessment of the tools used. For the level of CT and CDM, if a study did not report the level directly, we estimated it using the reported scores whenever data were available. We were unable to proceed with a meta-analysis of CT and CDM scores because the included studies used different measurement tools, yielding disparate findings. However, we performed a meta-analysis of the commonly used instrument tools identified in the selected studies. Each study may have reported CT and CDM for the overall population or the level of either CT or CDM, depending on its objectives. Data from some countries may also have limited quality and representativeness.

### 2.5. Quality Assessment

A quality score, modified from the Newcastle–Ottawa Scale (NOS), was used to assess the quality of each included article [19]. Three review authors (N.H.Z., T.M.H., and W.S.R.H.) independently assessed the quality of each included study using the adapted NOS for cross-sectional studies [20]. Disagreements over the NOS scores were resolved through discussion among these three authors, and when necessary, consultation with the fifth and sixth review authors (N.S.R. and Z.M.). The NOS uses a “star system” to evaluate studies based on three broad perspectives: (1) the selection of study groups; (2) the comparability of groups; and (3) the ascertainment of exposure/outcome. The quality scores for cross-sectional studies are categorized as follows: (1) very good: 9–10 points; (2) good: 7–8 points; (3) satisfactory: 5–6 points; and (4) unsatisfactory: 0–4 points. The detailed criteria for NOS assessment are presented in Supplementary Table S3.

### 2.6. Statistical Analysis

Statistical analyses were performed using R version 4.4.3. For meta-analyses, to estimate the pooled CT and CDM scores measured by the most common tools used across multiple studies, we used the “meta” package [21,22] and the “metamean” package [23] in R. We first imported the included studies into R and performed any necessary data cleaning and processing. In our meta-analyses, we chose a random-effects model due to the observed heterogeneity among the included studies. This model accounts for within-study variation in effect sizes, providing a more conservative estimate of the pooled effect size [22]. We also used the forest function to generate forest plots of the individual study effect sizes and their 95% confidence intervals (CIs), including a horizontal line representing the overall effect estimate. Additionally, to assess heterogeneity, we employed the I-squared (I^2^) statistic to quantify the proportion of total variation in effect sizes attributable to heterogeneity beyond chance. Publication bias of the meta-analyses could not be assessed due to the limited number of included studies. Publication bias tests are underpowered, and a visual inspection of a funnel plot may be misleading with such a small number of studies.

## 3. Results

**Figure 2:**
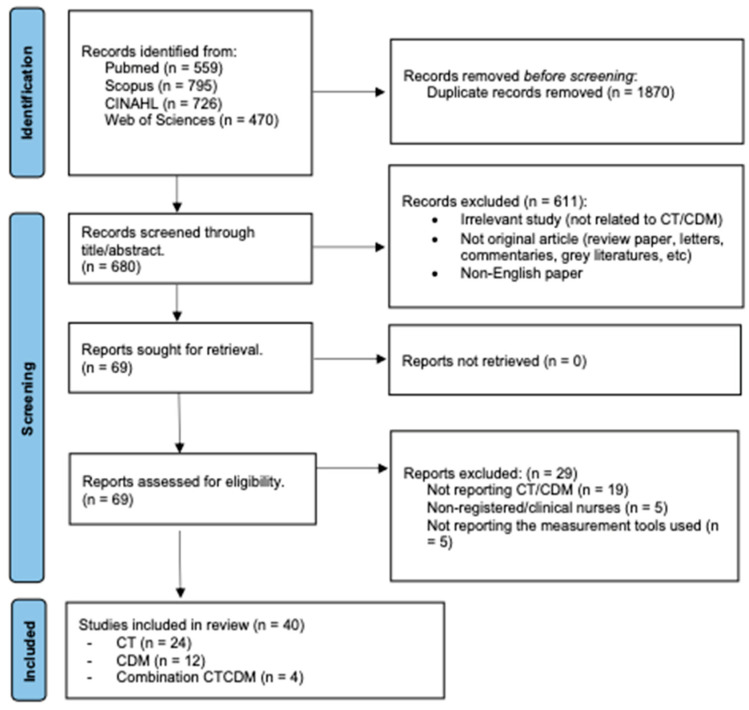
This is a flow diagram of the published articles evaluated for inclusion in this review.

A total of 2550 studies were identified through the database search. Figure 2 illustrates the flow of information through the identification and screening phases of the systematic review. A total of 680 studies were screened for eligibility through title and abstract screening, followed by full-text screening, ultimately yielding a total of 40 studies for review and synthesis. The NOS rated three studies as being of very good quality, thirty studies as being of good quality, and six as being of satisfactory quality. Only one selected study was rated as unsatisfactory. The detailed quality assessment of each study is presented in Table 1.

### 3.1. Characteristic of Included Studies

**Figure 3:**
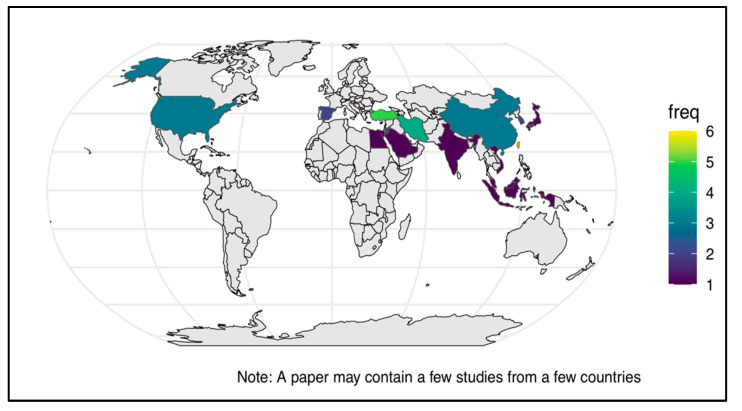
World map showing the distribution and frequency of CT/CDM studies.

**Figure 4:**
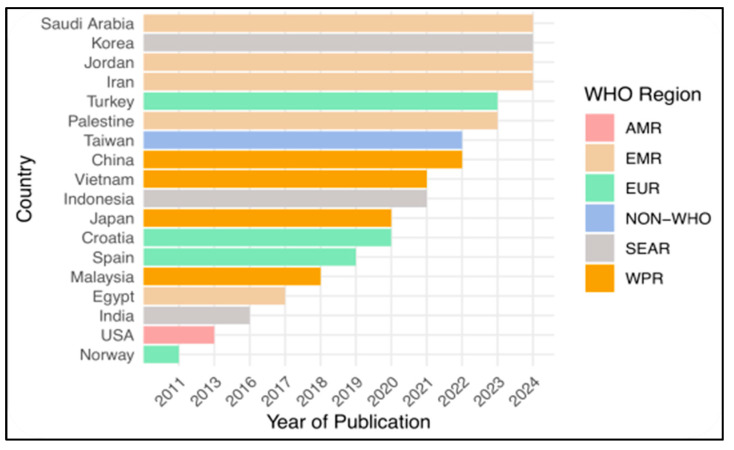
The latest year of publication by country and WHO region.

**Figure 5:**
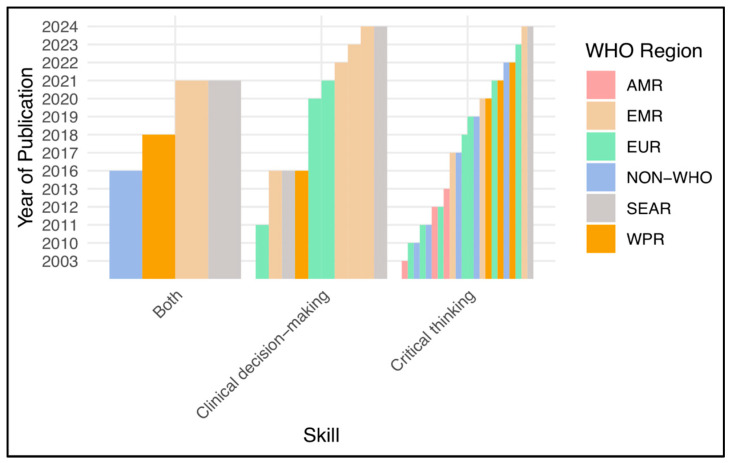
The latest year of publication by skill and WHO region.

Of the forty studies, twenty-four reported on CT skills, twelve reported on CDM skills, and the remaining four studies reported on a combination of both CT and CDM skills. Figure 3 presents a world map illustrating the geographical distribution of the selected studies at the country level and their publication frequency. Notably, these studies encompass a wide representation of World Health Organization (WHO) regions, including the Region of the Americas (three studies), the European Region (eleven studies), Southeast Asia (four studies), the Western Pacific (six studies), and the Eastern Mediterranean Region (ten studies). Taiwan (six studies) is classified as a non-member of the WHO due to the complex political issues affecting its international recognition [61,62]. Figure 4 shows the most recent year of publication by country and WHO region, highlighting Saudi Arabia, Korea, Jordan, and Iran as locations with the most recent publications. The regions with the most robust publication activity in CT and CDM in recent years are the Eastern Mediterranean Region, the Southeast Asia Region, and the European Region. Figure 5 shows a significant increase in publications on CT, CDM, and combined CT/CDM over the past decade. Studies with similarly themed components were grouped for narrative synthesis. Data were grouped based on the type of observed skill, the measurement tool used, and the predictive factors influencing the skill.

### 3.2. The Type of Observed Skills in the Selected Studies

Supplementary Material Table S4 summarizes the included studies that assessed CT, CDM, and both CT and CDM among nurses in clinical practice. Regarding CT skills, the studies varied in sampling method, sample size, type of hospital, response rate, and data analysis. The selected studies reporting on CT skills represented the following regions: the Americas (USA), Europe (Norway, Turkey, and Spain), the Western Pacific (China, Japan, and Vietnam), the Eastern Mediterranean (Egypt and Iran), Southeast Asia (South Korea), and a non-WHO region (Taiwan). Approximately twenty-three studies used a cross-sectional design, and one was an interventional study. Concerning sample size, 12 studies reported sample size calculations, while 12 others did not. Regarding hospital type, eight studies were conducted in teaching or university hospitals, three in medical centers, three in tertiary hospitals, one in a military hospital, seven in general and public hospitals, one in a private hospital, and one in general and teaching hospitals. Meanwhile, twenty studies reported response rates greater than 60%, and four studies reported response rates less than 60%. Regarding data analysis, this review found that most studies used descriptive and inferential statistics. Twenty studies used reliability analysis to assess the reliability of the tools, and four studies did not report it.

For CDM skills, the included studies varied in terms of hospital type, response rate, and data analysis methods. The selected studies reporting on CDM skills represented the Eastern Mediterranean Region (Jordan, Saudi Arabia, Iran, and Palestine), the European Region (Norway, Croatia, and Turkey), the Western Pacific Region (China), and the Southeast Asian Region (Korea and India). Eleven studies used a cross-sectional design, and one used a correlational design. Regarding sample size, six studies reported sample size calculations, while six others did not. Concerning hospital type, one study was conducted across three types of hospitals (educational, regional, and local). Two studies were conducted in general and private hospitals. Three were conducted in university hospitals, one in a tertiary-care hospital, four in government hospitals, and one in a pandemic hospital. Ten studies reported response rates greater than 60%, and two reported response rates less than 60%. Regarding data analysis, this review found that most studies used descriptive and inferential statistics, as well as reliability analysis.

In this review, we found that studies involving the combination of CT and CDM skills varied in terms of sampling methods, sample size calculations, and the types of hospital. The selected studies that reported on both CT and CDM skills represented the Western Pacific Region (Malaysia), the Southeast Asian Region (Indonesia), the Eastern Mediterranean Region (Jordan), and a non-WHO region (Taiwan). Three studies used a cross-sectional design, and one used a correlational design. All studies reported response rates greater than 60%. Regarding sampling methods, two studies used purposive sampling, one used convenience sampling, and the other used simple random sampling. Three studies reported sample size calculations, while one did not. In terms of hospital types, one study was conducted in two types of hospital: a medical center and a local hospital. The other three studies were conducted in a general hospital, a tertiary hospital, and a university hospital. For data analysis, this review found that all studies used descriptive and inferential statistics, as well as reliability analysis.

### 3.3. The Results of the Observed Skills and Predictive Factors

The included studies with complete objectives, results of the observed skills, and information on predictive factors are presented in Supplementary Table S5. For CT skills, this review revealed that four studies showed low or negative disposition or a poor level of CT, ten studies showed medium or average or moderate or middle or satisfactory or partially developed CT skills, two studies showed a high or positive level of CT, and eight studies did not explicitly report any level of CT skills. Regarding predictive factors, seventeen studies reported individual-level predictive factors, including gender, age, education, experience, clinical ladder, certification, on-the-job training (course, seminar, or continuous professional development), day-time shift, race, duration of working, position, and knowledge readiness. Meanwhile, four studies reported group-level predictive factors, including emergency department, educational hospital type, community health care, and critical care unit.

Regarding CDM skills, this review revealed that two studies reported a low level of CDM, two studies reported a high or good level of CDM, and the remaining eight did not explicitly report any level of CDM skills. Concerning predictive factors, ten studies reported individual-level predictors, including age, experience, education, gender, self-esteem, self-efficacy, locus of control, situation, working hours, and total structural empowerment. Only one study reported group-level predictive factors, specifically the surgical unit.

Meanwhile, regarding the combination of CT and CDM skills, we found the following: one study reported high levels of both CT and CDM skills among nurses in clinical practice; one study reported moderate CT skills and good CDM skills; one study reported low CT skills with intuitive decision making; and one study reported intuitive–analytical types of CDM. Regarding predictive factors, three studies reported factors at the individual levels, including knowledge readiness, experience, age, gender, ethnicity, and education. One study reported group-level predictive factors, specifically the unit.

### 3.4. The Measurement Tools Used in Selected Studies

A summary of the measurement tools used to assess CT and CDM skills among registered nurses in clinical practice is presented in Supplementary Material Table S6. The selected studies that reported on CT skills used a variety of tools, and several studies used more than one tool [30,32]. Ten measurement tools were identified in this review, including the California Critical Thinking Skills Test (two studies), the Nursing Critical Thinking in Clinical Practice Questionnaire (five studies), the Watson–Glaser Critical Thinking Appraisal (two studies), the California Critical Thinking Disposition Inventory (twelve studies), the Japanese Critical Thinking Disposition Scale (one study), the Decision Analytic Questionnaire (one study), the Health Sciences Reasoning Test (1one study), the Critical Thinking Questionnaire (one study), the Yoon Critical Thinking Disposition Scale (one study), and the Learning Transfer Tool (one study). Regarding reliability and psychometric properties, approximately twenty selected studies reported on reliability, but only four studies reported complete psychometric properties, including exploratory factor analysis (EFA) and confirmatory factor analysis (CFA).

Meanwhile, the selected studies that reported on CDM skills used multiple tools. A total of four measurement tools were identified in this review: the Clinical Decision Making in Nursing Scale (six studies), the 24-item Nursing Decision Making Instrument (four studies), the 36-item Questionnaire of Factors Affecting CDM (one study), and the Practical Knowledge Inventory for Nurses (one study). Regarding reliability and psychometric properties, all 12 selected studies reported only reliability data, without mentioning any other psychometric properties.

We found that all four selected studies reported on both CT and CDM skills but used different versions of measurement tools. A total of six measurement tools were identified across these studies, including multiple versions of the California Critical Thinking Disposition Inventory (two studies), the Critical Thinking Questionnaire (one study), the Critical Thinking Self-Assessment Scale (one study), multiple versions of the Clinical Decision-Making Model Inventory (CDMI or NDMI) (two studies), the Clinical Decision-Making Questionnaire (one study), and the Clinical Decision-Making Nursing Scale (one study). Regarding reliability and other psychometric properties, all four selected studies reported reliability data only; no other psychometric properties were reported.

### 3.5. The Results of a Meta-Analysis

From the summary of measurement tools used in the selected studies, we found that four tools were commonly used: the California Critical Thinking Disposition Inventory (CCTDI), the Nursing Critical Thinking Performance Scale (NCT4P), the Clinical Decision-Making Nursing Scale (CDMNS), and the Nursing Decision-Making Instrument (NDMI). Therefore, meta-analyses were conducted to derive conclusions about each of these tools.

**CCTDI:**

**Figure 6:**
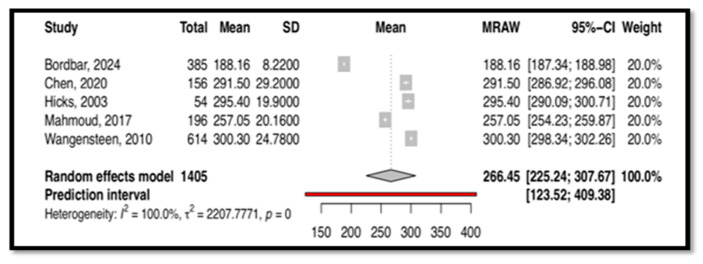
This is the forest plot of overall critical thinking score measured by CCTDI [26,29,32,37,43].

A total of five studies with 1405 subjects measured CT using the CCTDI scale. Among the included studies, one study utilized a Chinese version of the CCTDI [29], another employed an Arabic version [37], a Norwegian version was used in one study [43], and the original version was used in two studies [26,32]. The meta-analysis results, as presented in Figure 6, revealed significant heterogeneity (I^2^ = 100%). Thus, the meta-analysis was based on a random-effects model. The findings indicated an ambivalent overall disposition for CT with a mean score of 266.45 and a 95% confidence interval of (225.24; 307.67). This result was statistically significant (*p* < 0.05).

**NCT4P:**

**Figure 7:**
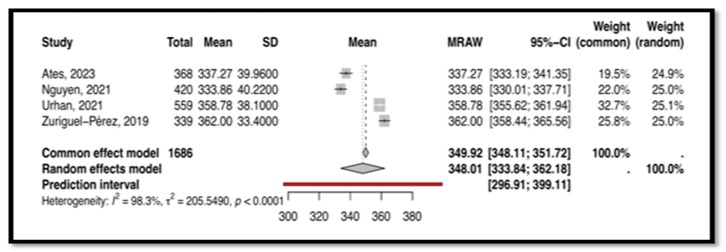
This is the forest plot of the overall critical thinking score measured by NCT4P [25,38,42,45].

Four studies, including 1686 subjects, provided data for pooling to assess CT as measured by the NCT4P. These studies utilized different versions of the NCT4P, including the Vietnamese version [38], the Turkish version [25,42], and the original Spanish version [45]. Heterogeneity analysis revealed significant heterogeneity between the included studies (I^2^ = 98.3%), as demonstrated in Figure 7. Therefore, a random-effects model was employed for the meta-analysis. The results indicated a moderate overall score, with a mean of 348.01 and a 95% confidence interval of (333, 362), which was statistically significant (*p* < 0.0001).

**CDMNS:**

**Figure 8:**
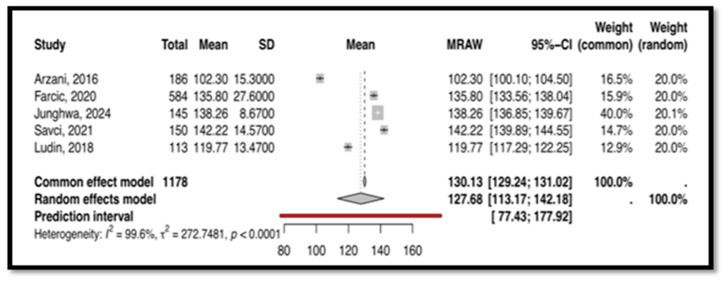
This is the forest plot of the overall clinical decision-making score measured by CDMNS [7,49,52,53,56].

Five selected studies with 1178 subjects measured CDM using the CDMNS tool. These studies used different versions of the CDMNS, including the original version [49,53], the Croatian version [52], the Turkish version [56], and the Malay version [7]. For this tool, the heterogeneity analysis indicated significant heterogeneity between the included studies (I^2^ = 99.6%), as demonstrated in Figure 8. Therefore, a random-effects model was employed, with the mean score being 127.68 and the 95% confidence interval ranging from 113 to 142. This result was statistically significant (*p* < 0.0001).

**NDMI:**

**Figure 9:**
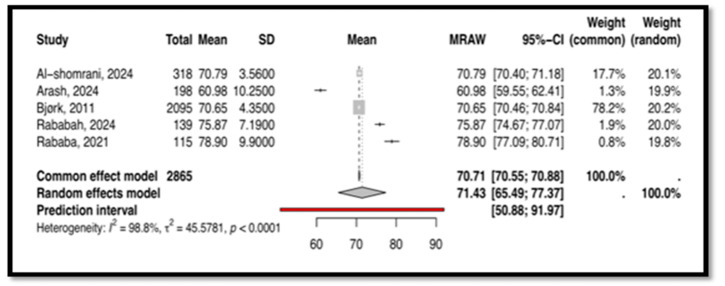
This is the forest plot of the overall clinical decision-making score measured by NDMI [47,48,51,55,60].

Another tool, the Nursing Decision-Making Instrument (NDMI) with the English original version, was used in five studies with 2865 subjects [47,48,51,55,60]. For this tool, the heterogeneity analysis indicated significant heterogeneity between the included studies (I^2^ = 98.8%), as demonstrated in Figure 9. Therefore, a random-effects model was employed, with the mean score being 71.43 and the 95% confidence interval ranging from 65.49 to 77.37. This result was statistically significant (*p* < 0.0001).

## 4. Discussion

To our knowledge, this study represents the first systematic review to comprehensively examine both CT and CDM skills among RNs in clinical practice. Most studies were conducted in the WHO region, and an increasing trend was observed in the number of studies assessing both CT and CDM skills after 2016. Regarding the focus of the studies, 60% assessed CT skills, 30% assessed CDM skills, and 10% assessed both CT and CDM skills. In terms of study design and settings, a significant proportion of studies lacked crucial information. For instance, 50% of CT studies, 50% of CDM studies, and 25% of studies assessing both CT and CDM did not report sample size calculations. The absence of sample size calculations hinders the evaluation of whether the study samples were adequately powered to draw valid and generalizable conclusions [63]. Inadequate sample sizes can lead to biased or unreliable results, potentially affecting the external validity of the findings [64]. Furthermore, 16.7% of CT studies and 16.7% of CDM studies reported low response rates. Consistent with previous reviews [14,65], this review also highlights the lack of a universally accepted conceptual framework for describing and evaluating CT and CDM skills in clinical practice.

Our findings revealed inconsistent results across studies for measuring CT and CDM skills, aligning with previous research. A scoping review of CT studies in 2015, a systematic review of CT in 2015, and a systematic review of CDM in 2021 all reported inconsistent findings, even when standardized tools were used, raising concerns about the reliability of these instruments in the nursing context [14,66,67]. This inconsistency may be attributable to the lack of a universally accepted conceptual model and standardized measurement tools, making it challenging to generalize results across studies. Regarding the predictive factors of CT and CDM skills, the selected studies suggested that both individual-level and group-level factors exert an influence on these skills [31,42,45,51,58]. However, no study in our review examined these factors using appropriate analytical methods, such as multilevel analysis. Multilevel analysis is particularly valuable when investigating how group-level factors influence individual-level outcomes, as it accounts for the hierarchical structure of the data (e.g., nurses nested within hospitals). Traditional single-level analyses of multilevel data can lead to misleading standard errors and significance tests [68,69,70].

This review highlights several key findings regarding the assessment of CT and CDM among nurses in clinical practice. Firstly, a significant limitation identified was the lack of sensitivity of many currently available standard instruments in measuring CT and CDM in the specific context of nursing practice. While tools like the Nursing Critical Thinking Questionnaire in Clinical Practice and the 24-item Nursing Decision-Making Instrument were developed specifically for this context [71,72], their widespread adoption and rigorous psychometric evaluation are still needed. Secondly, a concerning observation was the inconsistent and variable reporting of reliability and validity across the included studies. For CT, while several commercially available tools were utilized, their cost may pose a barrier to routine evaluation in clinical settings. Furthermore, some studies failed to adequately test the reliability of these tools within their specific context. Only four studies comprehensively reported on the reliability and psychometric properties of their chosen instruments. Similarly, for CDM and combined assessments of CT and CDM, a notable absence of comprehensive reporting on the psychometric properties of the measurement tools was observed. It is crucial to emphasize that rigorous psychometric testing, including reliability and validity assessments, is a fundamental requirement for any measurement tool used in clinical practice, research, or evidence-based projects. This ensures the quality, accuracy, and ethical conduct of such endeavors.

Psychometric testing often involves the use of both EFA and CFA as essential components of the validation process for measurement instruments. EFA is typically used to explore the underlying factor structure of the items in the instrument. Meanwhile, CFA is a subsequent step to confirm the hypothesized factor structure of the instrument [73,74]. To discuss the model fit of CFA, we should consider the criteria of various model fit indices. It has been suggested that for absolute fit, a Chi-square p-value greater than 0.05, Root Mean Square Error of Approximation (RMSEA) values less than 0.08, and Goodness of Fit Index (GFI) greater than 0.90 are acceptable; for an incremental fit, the Adjusted Goodness of Fit (AGFI), Comparative Fit Index (CFI), and Tucker-Lewis Index (TLI) should be greater than 0.9 for a good fit; and for a parsimonious fit, the Chi-square/df value should be less than 3.0 [75]. Based on the absolute fit indices, all four studies that reported conducting CFA demonstrated acceptable absolute fit. However, for an incremental fit, some studies exhibited an inadequate fit with CFI = 0.73, TLI = 0.72 [38], AGFI = 0.71 [6], CFI = 0.629, and TLI = 0.621 [45,46]. These findings suggest that the model may not adequately capture the relationships between latent factors and observed variables [75,76]. Meanwhile, for studies examining CDM and combinations of CT and CDM, none conducted factor analyses to establish validity. This lack of validation raises concerns about the measurement properties of the instruments used in these studies.

This meta-analysis, the first of its kind, synthesizes findings on CT and CDM tools used among nurses. Four tools emerged as commonly used: CCTDI, NCT4P, CDMNS, and NDMI. The CCTDI pooled CT score was 266.45 (95% CI: 225.24; 307.67), suggesting an “ambivalent” disposition. High heterogeneity (I^2^ = 100%) limited the generalizability of this finding. The NCT4P pooled CT score was 348.01 (95% CI: 333; 362), indicating “moderate” CT. High heterogeneity (I^2^ = 98.3%) also limited the generalizability of this finding. Meanwhile, the CDMNS pooled CDM score was 127.68 (95% CI: 113; 142) with high heterogeneity (I^2^ = 99.6%), and the NDMI pooled CDM score was 71.43 (95% CI: 65.49; 77.37) with high heterogeneity (I^2^ = 98.8%). Overall, high heterogeneity across all tools (CCTDI, NCT4P, CDMNS, NDMI) limited the generalizability of the findings. This variability may be attributed to differences in participant characteristics, study design, and instrument versions.

Due to the limited number of studies included in our meta-analyses, a formal assessment of publication bias was not feasible. Tests for publication bias are underpowered with small sample sizes, and visual inspection of a funnel plot for asymmetry can be unreliable in such cases. The substantial degree of heterogeneity observed across all meta-analyses, as evidenced by high I^2^ values, indicates an extremely high level of variability among the included studies. While meta-analysis offers the potential to investigate sources of heterogeneity, the presence of such high heterogeneity underscores the need for cautious interpretation of the pooled results [77]. Researchers should carefully consider potential sources of heterogeneity, such as differences in study methodologies, participant characteristics, interventions, and the specific instruments used [78,79,80]. These factors can significantly influence the observed effect sizes and should be explored in future research to better understand the variability in the literature.

The findings of this review carry important implications for both nursing practice and education. The observed variability in CT and CDM skills among RNs highlights the need for ongoing assessment in clinical settings to identify nurses who may benefit from targeted professional development. The predictive factors identified, such as experience, education, and on-the-job training, suggest potential avenues for intervention. For instance, healthcare organizations could invest in mentorship programs for novice nurses and ensure access to continuous education focused on enhancing these critical skills. Furthermore, the lack of consistent use and comprehensive psychometric evaluation of measurement tools underscores a need for the development and validation of instruments specifically tailored to the nursing context. For nursing education, these findings suggest a continued emphasis on integrating critical thinking and decision-making skills development throughout curricula, potentially tailoring approaches based on educational level to foster stronger foundational abilities.

### Limitations

Several important limitations to this review should be acknowledged, which may limit the generalizability of the findings. Firstly, some included studies reported low response rates or lacked sample size calculations, potentially limiting the overall representativeness of the findings. Secondly, publication bias is an inherent concern in any systematic review. We endeavored to address this issue by conducting a comprehensive search across multiple databases, including electronic databases, reference lists, and by contacting study authors for additional information. Thirdly, as with any systematic review, it is challenging to obtain complete data relevant to all research objectives. Our review also faced limitations in the data available from several selected studies, particularly regarding sample size calculations, scoring systems, and the reporting of reliability and psychometric properties, including EFA and CFA data. Fourthly, the use of diverse measurement tools across the included studies contributed to high heterogeneity, which may have impacted the quality of the evidence. However, only one out of forty papers was rated as unsatisfactory, providing a reasonable evidence base for drawing conclusions. To improve future research, more robust methodologies are needed, including comprehensive descriptions of research methods and the incorporation of reference standards. Furthermore, research teams should ideally include statisticians and experts in experimental design to ensure rigorous evaluation of both CT and CDM skills in clinical practice. A better understanding of research methodologies and analysis will empower researchers and administrators to develop more effective frameworks, strategies, and interventions to enhance nurses’ CT and CDM skills.

## 5. Conclusions

This systematic review and meta-analyses provide an overview of CT and CDM skills among RNs in clinical practice. In summary, the results of the current review indicate that there is significant variability in the findings regarding the level of CT and CDM skills among registered nurses. This variability underscores the need for ongoing assessment in clinical settings to inform targeted professional development. The identified predictive factors also offer insights for potential interventions in both practice (e.g., mentorship, training) and education (e.g., curriculum enhancement). The complexity of these constructs, coupled with the lack of a universally accepted conceptual model and standardized measurement tools, has resulted in difficulties in generalizing the findings across studies. Additionally, both individual-level and group-level predictive factors have been identified as influencing CT and CDM skills. Future research should prioritize the implementation of high-quality research methodologies, the development of new instruments with robust psychometric properties, and the application of advanced statistical techniques such as multilevel modeling to better understand the influence of hierarchical factors on these skills. This will enhance the generalizability and clinical relevance of the findings.

## Figures and Tables

**Table 1:** Characteristics of the included studies.

Study ID	Measurement Tools Used			Findings			Quality Assessment (NOS)			
				Score Indicators	Predictive Factors Influenced the Study		S	C	O	Total Score
	Instrument	Reliability Test	Psychometric Test		Individual	Group				
Ali-Abadi, 2020 [24]	CCTST-FB	Available	N/A	CT level	Available	N/A	***	**	**	7 (Good studies)
Ates, 2023 [25]	CTSiCPfN	Available	N/A	CT skill	Available	N/A	****	*	**	7 (Good studies)
Bordbar, 2024 [26]	CCTDI	Available	N/A	CT disposition	Available	N/A	****	**	***	9 (Very good studies)
Chang, 2011 [27]	WGCTA-CV	Available	N/A	CT ability	Available	N/A	***	**	**	7 (Good studies)
Chen, 2019 [28]	TCTDI	Available	N/A	CT disposition	Available	N/A	****	*	**	7 (Good studies)
Chen, 2020 [29]	CTDI-CV	N/A	N/A	CT disposition	N/A	N/A	****	*	**	7 (Good studies)
Feng, 2010 [30]	Tw-WGCT; TCTDI	Available	N/A	CT competence, CT disposition	Available	N/A	****	**	**	8 (Good studies)
Futami, 2020 [31]	JCTDS	Available	N/A	CT disposition	Available	Available	***	**	**	7 (Good studies)
Hicks, 2003 [32]	CCTDI, CCTST, DAQ	N/A	N/A	CT disposition, CT skill, CDM consistency	N/A	N/A	**	*	**	5 (Satisfactory studies)
Hsu, 2017 [33]	CTD	Available	N/A	CT disposition	Available	N/A	***	**	**	7 (Good studies)
Kaya, 2012 [34]	CCTDI	Available	N/A	CT disposition	Available	N/A	***	**	**	7 (Good studies)
Lang, 2013 [35]	HSRT	N/A	N/A	CT skills	Available	N/A	****	**	**	8 (Good studies)
Lee, 2022 [36]	CTQ	Available	N/A	CT ability	Available	N/A	***	**	**	7 (Good studies)
Mahmoud, 2017 [37]	CCTDI	Available	N/A	CT disposition	N/A	N/A	****	*	**	7 (Good studies)
Nguyen, 2021 [38]	N-CT-4 P	Available	Available	CT ability	Available	N/A	*****	**	**	9 (Very good studies)
Park, 2024 [39]	CTDS	Available	N/A	CT disposition	N/A	N/A	****	*	***	8 (Good studies)
Polat, 2019 [6]	Turkish CCTDI	Available	Available	CT disposition	Available	N/A	*****	*	**	8 (Good studies)
Schubert, 2012 [40]	LTT	N/A	N/A	CT skill	N/A	N/A	**		**	4 (Unsatis-factory studies
Sun, 2022 [41]	CTDI-CV	Available	N/A	CT disposition	Available	N/A	***	*	**	6 (Satisfactory studies)
Urhan, 2021 [42]	N-CT-4 P-Tv	Available	N/A	CT skill	Available	Available	***	**	**	7 (Good studies)
Wangen-steen, 2010 [43]	CCTDI	Available	N/A	CT disposition	Available	N/A	***	**	**	7 (Good studies)
Wangen-steen, 2011 [44]	CCTDI	Available	N/A	CT disposition	N/A	N/A	***		**	5 (Satisfactory studies)
Zuriguel-Pérez, 2019 [45]	N-CT-4 P	Available	Available	CT level	Available	N/A	****	**	**	8 (Good studies)
Zuriguel-Pérez, 2018 [46]	N-CT-4 P	Available	Available	CT level	Available	N/A	****	**	**	8 (Good studies)
Abu Arra, 2023 [10]	CDMNS	Available	N/A	CDM level	Available	N/A	****	**	**	8 (Good studies)
Alshomrani, 2024 [47]	NDMI	Available	N/A	Decision-making style	Available	N/A	****	**	**	8 (Good studies)
Arash, 2024 [48]	NDMI	Available	N/A	Decision-making style	Available	N/A	****	**	**	8 (Good studies)
Arzani, 2016 [49]	CDMNS	Available	N/A	CDM level	Available	N/A	***	**	**	7 (Good studies)
Batran, 2022 [50]	CDMNS	Available	N/A	CDM level	N/A	N/A	****	*	**	7 (Good studies)
Bjørk, 2011 [51]	NDMI	Available	N/A	Decision-making style	Available	Available	**	**	**	6 (Satisfactory studies)
Farčić, 2020 [52]	CDMNS	Available	N/A	CDM level	Available	N/A	**	**	**	6 (Satisfactory studies)
Junghwa Yun, 2024 [53]	CDMNS	Available	N/A	CDM level	N/A	N/A	****	*	***	8 (Good studies)
Nageshwar, 2016 [54]	36-item Q	Available	N/A	N/A	Available	N/A	***	*	**	6 (Satisfactory studies)
Rababah, 2024 [55]	NDMI	Available	N/A	Decision-making style	Available	N/A	****	**	***	9 (Very good studies)
Savci, 2021 [56]	CDMNS	Available	N/A	CDM level	Available	N/A	***	**	**	7 (Good studies)
Wu, 2016 [57]	PKIN	Available	N/A	Nurses’ ability levels	Available	N/A	***	**	**	7 (Good studies)
Chen, 2016 [58]	CCTDI, CDMI	Available	N/A	CT disposition, CDM abilities	Available	Available	***	**	**	7 (Good studies)
Dewi, 2021 [59]	CTQ, CDMQ	Available	N/A	CT ability, CDM ability	N/A	N/A	****	*	**	7 (Good studies)
Ludin, 2018 [7]	SF-CTDI-CV, CDMNS	Available	N/A	CT disposition, CDM level	Available	N/A	****	**	**	8 (Good studies)
Rababa, 2021 [60]	CTSAS, NDMI	Available	N/A	CT skills, CDM modes	Available	N/A	****	**	**	8 (Good studies)

NOS: Newcastle-Ottawa Scale (S: selection, C: comparability, and O: outcome); N/A: Not available; CT: Critical thinking; CDM: Clinical decision-making; CCTST-FB (The California Critical Thinking Skills Test Form-B); CTSiCPfN/ N-CT-4 P-Tv: Turkish version of The Nursing Critical Thinking in Clinical Practice; CCTDI: California Critical Thinking Disposition Inventory; WGCTA-CV: Chinese Watson-Glaser Critical Thinking Appraisal; TCTDI: Yeh Taiwan Critical Thinking Disposition; CTDI-CV: Chinese Version of Critical Thinking Disposition Inventory; Tw-WGCT: Taiwan Watson -Glaser Critical Thinking Appraisal; JCTDS: Japanese Critical Thinking Disposition Scale; CCTST: California Critical Thinking Skills Test; DAQ: Decision Analytic Questionnaire; CTD: Critical Thinking Disposition; HSRT: Health Sciences Reasoning Test; CTQ: Critical thinking questionnaire; N-CT-4 P: Nursing Critical Thinking in Clinical Practice; CTDS: Critical Thinking Disposition Scale; Turkish CCTDI: Turkish California Critical Thinking Disposition Inventory; LTT: Learning Transfer Tool; CDMNS: Clinical Decision Making in Nursing Scale; NDMI/CDMI: Nursing Decision Making Instrument; 36-item Q: 36-item Questionnaire of Factors Affecting CDM; PKIN: Practical Knowledge Inventory for Nurses; CTQ: Critical Thinking Questionnaire; CDMQ: Clinical Decision-making Questionnaire; SF-CTDI-CV: Short Form-Critical Thinking Disposition Inventory-Chinese Version; CTSAS: Critical Thinking Self-Assessment Scale. ‘*’ indicates a point awarded for a specific criterion that meets a certain quality standard.

## Data Availability

All relevant data are within the paper and its Supporting Information files. Codes can be found here: https://github.com/eidaadz/SRMA_CTCDM (accessed on 25 December 2024). The corresponding authors can be contacted for more information.

## Supplementary Materials

The following supporting information can be downloaded at: https://www.mdpi.com/article/10.3390/nursrep15050175/s1, S1: PRISMA checklist; Table S1: Search terms; Table S2: Data extraction form; Table S3: NOS assessment; Table S4: The observed skills; Table S5: The finding score and predictive factors; Table S6: The measurement tools.

## Public Involvement Statement

No public involvement in any aspect of this research.

## Guidelines and Standards Statement

The manuscript followed the revised Preferred Reporting Items for Systematic Reviews and Meta-Analyses (PRISMA) 2020.
