# Supplementary material for: Critical Thinking and Clinical Decision Making Among Registered Nurses in Clinical Practice: A Systematic Review and Meta-Analysis
Source: Nurs Rep. 2025 May 20;15(5):175. doi: 10.3390/nursrep15050175 (PMC12113899; doi:10.3390/nursrep15050175)
Supplement: Supplementary file 1 [file nursrep-15-00175-s001.zip › nursrep-3628992-supplementary.pdf]

Appendix (Supplementary)

S1 PRISMA checklist

Section and Topic	Item #	Checklist item	Location where item is reported
TITLE			
Title	1	Identify the report as a systematic review.	Title page<br>Page 1
ABSTRACT			
Abstract	2	According to the PRISMA 2020 for Abstracts checklist.	Page 1
INTRODUCTION			
Rationale	3	Describe the rationale for the review in the context of existing knowledge.	Page 2
Objectives	4	Provide an explicit statement of the objective(s) or question(s) the review addresses.	Page 2
METHODS			
Eligibility criteria	5	Specify the inclusion and exclusion criteria for the review and how studies were grouped for the syntheses.	Page 3
Information sources	6	Specify all databases, registers, websites, organisations, reference lists and other sources searched or consulted to identify studies. Specify the date when each source was last searched or consulted.	Page 3
Search strategy	7	Present the full search strategies for all databases, registers and websites, including any filters and limits used.	Page 3
Selection process	8	Specify the methods used to decide whether a study met the inclusion criteria of the review, including how many reviewers screened each record and each report retrieved, whether they worked independently, and if applicable, details of automation tools used in the process.	Page 4
Data collection process	9	Specify the methods used to collect data from reports, including how many reviewers collected data from each report, whether they worked independently, any processes for obtaining or confirming data from study investigators, and if applicable, details of automation tools used in the process.	Page 4
Data items	10a	List and define all outcomes for which data were sought. Specify whether all results that were compatible with each outcome domain in each	Page 4

Section and Topic	Item #	Checklist item	Location where item is reported
		study were sought (e.g. for all measures, time points, analyses), and if not, the methods used to decide which results to collect.	
	10b	List and define all other variables for which data were sought (e.g. participant and intervention characteristics, funding sources). Describe any assumptions made about any missing or unclear information.	Page 4
Study risk of bias assessment	11	Specify the methods used to assess risk of bias in the included studies, including details of the tool(s) used, how many reviewers assessed each study and whether they worked independently, and if applicable, details of automation tools used in the process.	Page 4
Effect measures	12	Specify for each outcome the effect measure(s) (e.g. risk ratio, mean difference) used in the synthesis or presentation of results.	Page 5
Synthesis methods	13a	Describe the processes used to decide which studies were eligible for each synthesis (e.g. tabulating the study intervention characteristics and comparing against the planned groups for each synthesis (item #5)).	Page 4
	13b	Describe any methods required to prepare the data for presentation or synthesis, such as handling of missing summary statistics, or data conversions.	Page 4
	13c	Describe any methods used to tabulate or visually display results of individual studies and syntheses.	Page 5
	13d	Describe any methods used to synthesize results and provide a rationale for the choice(s). If meta-analysis was performed, describe the model(s), method(s) to identify the presence and extent of statistical heterogeneity, and software package(s) used.	Page 5
	13e	Describe any methods used to explore possible causes of heterogeneity among study results (e.g. subgroup analysis, meta-regression).	N/A
	13f	Describe any sensitivity analyses conducted to assess robustness of the synthesized results.	N/A
Reporting bias assessment	14	Describe any methods used to assess risk of bias due to missing results in a synthesis (arising from reporting biases).	N/A
Certainty assessment	15	Describe any methods used to assess certainty (or confidence) in the body of evidence for an outcome.	Page 5
RESULTS			
Study selection	16a	Describe the results of the search and selection process, from the number of records identified in the search to the number of studies included in the review, ideally using a flow diagram.	Page 5
	16b	Cite studies that might appear to meet the inclusion criteria, but which were excluded, and explain why they were excluded.	
Study characteristics	17	Cite each included study and present its characteristics.	Page 9
Risk of bias in studies	18	Present assessments of risk of bias for each included study.	Page 5
Results of individual studies	19	For all outcomes, present, for each study: (a) summary statistics for each group (where appropriate) and (b) an effect estimates and its precision (e.g. confidence/credible interval), ideally using structured tables or plots.	Page 6

Section and Topic	Item #	Checklist item	Location where item is reported
Results of syntheses	20a	For each synthesis, briefly summarise the characteristics and risk of bias among contributing studies.	Page 9
	20b	Present results of all statistical syntheses conducted. If meta-analysis was done, present for each the summary estimate and its precision (e.g. confidence/credible interval) and measures of statistical heterogeneity. If comparing groups, describe the direction of the effect.	Page 3
	20c	Present results of all investigations of possible causes of heterogeneity among study results.	N/A
	20d	Present results of all sensitivity analyses conducted to assess the robustness of the synthesized results.	N/A
Reporting biases	21	Present assessments of risk of bias due to missing results (arising from reporting biases) for each synthesis assessed.	N/A
Certainty of evidence	22	Present assessments of certainty (or confidence) in the body of evidence for each outcome assessed.	N/A
DISCUSSION			
Discussion	23a	Provide a general interpretation of the results in the context of other evidence.	Page 4
	23b	Discuss any limitations of the evidence included in the review.	Page 16
	23c	Discuss any limitations of the review processes used.	Page 16
	23d	Discuss implications of the results for practice, policy, and future research.	Page 17
OTHER INFORMATION			
Registration and protocol	24a	Provide registration information for the review, including register name and registration number, or state that the review was not registered.	Page 3
	24b	Indicate where the review protocol can be accessed, or state that a protocol was not prepared.	Page 18
	24c	Describe and explain any amendments to information provided at registration or in the protocol.	N/A
Support	25	Describe sources of financial or non-financial support for the review, and the role of the funders or sponsors in the review.	Page 17
Competing interests	26	Declare any competing interests of review authors.	Page 18

Section and Topic	Item #	Checklist item	Location where item is reported
Availability of data, code and other materials	27	Report which of the following are publicly available and where they can be found: template data collection forms; data extracted from included studies; data used for all analyses; analytic code; any other materials used in the review.	Page 18

Table S1: Search Terms

Databases	Search terms
Web of Science	TI= (“critical thinking” OR “clinical decision” OR “clinical reasoning” OR “clinical judgement”) AND TI= (nurse OR nurses OR nursing) NOT TI= (“student” OR “students” OR “student’s” OR “students” OR “education” OR “Course” OR “Courses” OR “Graduate” OR “Undergraduate” OR “Educator” OR “Educators” OR “Interns”)
PubMed	(("critical thinking"[Title] OR "clinical decision"[Title] OR "clinical reasoning"[Title] OR "clinical judgement"[Title]) AND ("nurse"[Title] OR "nurses"[Title] OR "nursing"[Title])) NOT ("student"[Title] OR "students"[Title] OR "student's"[Title] OR "students"[Title] OR "education"[Title] OR "Course"[Title] OR "Courses"[Title] OR "Graduate"[Title] OR "Undergraduate"[Title] OR "Educator"[Title] OR "Educators"[Title] OR Interns[Title])
Scopus	TITLE ("critical thinking" OR "clinical decision" OR "clinical reasoning" OR "clinical judgement") AND TITLE (nurse OR nurses OR nursing) AND NOT TITLE ("student" OR "students" OR "student's" OR "students" OR "education" OR "Course" OR "Courses" OR "Graduate" OR "Undergraduate" OR "Educator" OR "Educators" OR "Interns")

CINAHL	((" critical thinking in nursing "[Title] OR " clinical decision making in nursing "[Title] NOT “students or college students or higher education or further education or university students” [Title] NOT “clinical reasoning” [Title] NOT” clinical judgement” [Title]))

Table S2: Data extraction form

No	1st author, publication year	Country	Study design	Population	Type of Hospital	Aim of study	Data analysis	Measurement tool used								Findings			
																Results of the observed skills		Predictive factors influenced the	
								Instrument	Domains	Description of tool	Aim of tool	Scoring	Reliability	EFA	CFA	Level	Score	Individual	Group

Table S3: Newcastle-Ottawa Scale for quality assessment of selected studies

Components	Newcastle-Ottawa Quality Assessment Scale original version [1]	Newcastle-Ottawa Scale adapted for cross-sectional studies [2]
Selection	1) Representativeness of the exposed cohort<br>a) truly representative of the average (describe) in the community *<br>b) somewhat representative of the average in the community *<br>c) selected group of users e.g., nurses, volunteers<br>d) no description of the derivation of the cohort<br><br>2) Selection of the non-exposed cohort<br>a) drawn from the same community as the exposed cohort *<br>b) drawn from a different source<br>c) no description of the derivation of the non-exposed cohort	1) Representativeness of the sample:<br>a) Truly representative of the registered or clinical nurses * (all subjects or random sampling)<br>b) Somewhat representative of the average in the registered nurse's population* (non-random sampling)<br>c) Other than registered or clinical nurses (student nurse, nurse educators, nursing lecturer etc).<br>d) No description of the sampling strategy.<br><br>2) Sample size explanation must include the inclusion and exclusion criteria:<br>a) Justified and satisfactory. *<br>b) Not justified.

	3) Ascertainment of exposure<br>a) secure record (e.g., surgical records) *<br>b) structured interview *<br>c) written self-report<br>d) no description<br><br>4) Demonstration that outcome of interest was not present at start of study<br>a) yes *<br>b) no	3) Non-response rate:<br>a) The response rate is satisfactory *<br>b) The response rate is unsatisfactory<br>c) No description of the response rate<br><br>4) Ascertainment of the screening / surveillance tool<br>a) Fully validated measurement tool including reliability, EFA and CFA **<br>b) Non-fully validated measurement tool, but the tool is available or described at least for the reliability*<br>c) No description of the measurement tool.<br><br>(Maximum 5 stars)
Comparability	1) Comparability of cohorts on the basis of the design or analysis<br>a) study controls for (select the most important factor) *	1) The potential predictive factors were investigated.<br>a) The study investigates the potential predictive factors from either individual or group-level factor *<br>b) The study does not investigate potential predictive factors<br>2) The score of the skills were reported

	b) study controls for any additional factor * (This criteria could be modified to indicate specific control for a second important factor.)	a) The study investigates the score of the skills*<br>b) The study does not investigate the score of the skills<br><br>(Maximum 2 stars)
Outcome	1) Assessment of outcome<br>a) independent blind assessment *<br>b) record linkage *<br>c) self-report<br>d) no description<br><br>2) Was follow-up long enough for outcomes to occur<br>a) yes (select an adequate follow up period for outcome of interest) *<br>b) no<br><br>3) Adequacy of follow up of cohorts<br>a) complete follow up - all subjects accounted for *	1) Assessment of the outcome:<br>a) Independent blind assessment. **<br>b) Record linkage. **<br>c) Self report. **<br>d) No description<br><br>2) Statistical test:<br>a) The statistical test used to analyze the data is clearly described and appropriate *<br>b) The statistical test is not appropriate, not described or incomplete.<br><br>(Maximum 3 stars)

	b) subjects lost to follow up unlikely to introduce bias - small number lost - > ____ % (select an adequate %) follow up, or description provided of those lost) *<br>c) follow up rate < ____% (select an adequate %) and no description of those lost	

Table S4: The observed skills in selected studies

No	Author, Year	Country	Study design	Population	Sampling method	Sample size calculation	Type of Hospital	Aim of study	Response rate	Data analysis
Critical Thinking										
1	Ali-Abadi, 2020	Iran	Cross-sectional study	Clinical nurses	ICU: Census sampling; Medical-surgical: Random cluster sampling	N/A	Teaching hospital	To compare the level of CT in medical-surgical and ICU nurses and investigate the factors explaining it	ICU nurses: 100%; Medical-surgical nurse: 80%	Descriptive statistics; Reliability analysis; Inferential statistics (independent samples t-test, ANOVA, and regression analysis)
2	Ates, 2023	Turkey	Cross-sectional study	Clinical nurses	Convenience sampling	A post hoc test, demonstrating alpha .05 and 98% confidence interval (CI)	University hospital	To investigate the relationship between nurses' CT skills and job performance	Response rate: 100%	Descriptive statistics; Reliability analysis; Inferential statistics (Pearson correlation analysis, multiple linear regression)

No	Author, Year	Country	Study design	Population	Sampling method	Sample size calculation	Type of Hospital	Aim of study	Response rate	Data analysis
3	Bordbar, 2024	Iran	Cross-sectional study	Clinical nurses	Stratified random sampling	Calculated using a formula with an error level of 5%	Educational hospitals	To investigate the state of critical thinking and its effect on moral courage and moral sensitivity in nurses	Response rate: 100 %	Descriptive statistics; Reliability analysis; Inferential statistics (Pearson's correlation coefficient, T-test, ANOVA, multiple linear regression)
4	Chang, 2011	Taiwan	Cross-sectional and correlation research design	Clinical nurses	Convenience sampling	N/A	Medical centre	To examine the relationships between CT ability and nursing competence	Response rate: 72.6 %	Descriptive statistics; Reliability analysis; Inferential statistics (One-way ANOVA, Pearson's product–moment correlation, Stepwise hierarchical regression analysis)

No	Author, Year	Country	Study design	Population	Sampling method	Sample size calculation	Type of Hospital	Aim of study	Response rate	Data analysis
5	Chen, 2019	Taiwan	Cross-sectional study	Registered nurse	Stratified random sampling	Calculated using G*Power 3.1.9.2 with a two-tailed effect size as 0.30, with α = 0.05	Medical centre hospital	To examine whether professional qualifications would affect self-reflection and critical thinking in the experienced registered nurses	Novice RNs: 95.8%; 96.1%: experienced RNs	Descriptive statistics; Reliability analysis; Inferential statistics (Two-sample t-tests, Partial least squares structural equation modelling-PLS-SEM)
6	Chen, 2020	China	Cross-sectional study	Clinical nurses	Convenience sampling	Calculated using formula: N > 50 + 8m, m = number of independent variables = 5	Tertiary hospitals	To explore the relationship between critical thinking disposition and research competence among clinical nurses	Response rate: 100 %	Descriptive statistics; Inferential statistics (Pearson bivariate correlation, The linear regression analysis)
7	Feng, 2010	Taiwan	Cross-sectional study	Clinical nurses	Stratified random sampling	Randomly selected 10% to 15% of the population of each ladder group as the sample	General hospital	To measure the critical thinking competence, critical thinking disposition and to explore the related factors	Response rate: 100 %	Descriptive statistics; Reliability analysis; Inferential statistics (Chi-square test,

No	Author, Year	Country	Study design	Population	Sampling method	Sample size calculation	Type of Hospital	Aim of study	Response rate	Data analysis
										ANOVA and Scheffe tests)
8	Futami, 2020	Japan	Cross-sectional study	Registered nurse	Cluster sampling	N/A	General hospital; Teaching hospital	To explore the organizational and personal factors related to critical thinking disposition	Nurses: 73.7%; Nurse managers: 93.2%	Descriptive statistics; Reliability analysis; Inferential statistics (T-test, Pearson's correlation coefficient, ANOVA, Multiple regression analysis)

No	Author, Year	Country	Study design	Population	Sampling method	Sample size calculation	Type of Hospital	Aim of study	Response rate	Data analysis
9	Hicks, 2003	USA	Cross-sectional correlational study	Registered nurse	Cluster sampling	N/A	Private teaching hospital	To examine the relationship among the concepts of clinical knowledge, critical thinking dispositions and skills, and decision-making consistency across decision tasks of varying complexity	Response rate: 47 %	Descriptive statistics; Reliability analysis; Inferential statistics (multiple and logistic regression)
10	Hsu, 2017	Taiwan	Cross-sectional study	Nurse practitioner	Purposive sampling	N/A	General hospital	To explore the CTD of nurse practitioners and related factors	Response rate: 94 %	Descriptive statistics; Reliability analysis; Inferential statistics (Correlation analysis, t test, ANOVA, Multiple regression analysis)

No	Author, Year	Country	Study design	Population	Sampling method	Sample size calculation	Type of Hospital	Aim of study	Response rate	Data analysis
11	Kaya, 2012	Turkey	Cross-sectional study	Emergency nurses	Cluster sampling	N/A	Educational hospital	To determine the critical thinking dispositions of emergency nurses and any influencing factors related to critical thinking	Response rate: 63.6 %	Descriptive statistics; Reliability analysis; Inferential statistics (one-way ANOVA, independent samples test and Pearson Correlation)
12	Lang, 2013	USA	Cross-sectional study	Registered nurse	Random sampling	Using an established minimum sample size by Cohen (1992)	Military hospital	To examine critical thinking skills among registered nurses who work in a military hospital	Response rate: 97 %	Descriptive statistics; Inferential statistics (Regression analysis, ANOVA)
13	Lee, 2022	Taiwan	Cross-sectional study	Intensive care unit nurses	Purposive sampling	N/A	General medical hospital	To explore the effectiveness of a digital learning management system in enhancing intensive care unit nurses'critical care knowledge and critical thinking tendency	Response rate: 96 %	Descriptive analysis; Reliability analysis; Inferential statistics (Independent T test, One-way ANOVA, Pearson correlation)

No	Author, Year	Country	Study design	Population	Sampling method	Sample size calculation	Type of Hospital	Aim of study	Response rate	Data analysis
14	Mahmoud, 2017	Egypt	Cross-sectional study	Clinical nurses	Systematic random sampling	Calculated using Slovin's formula with a 95% confidence level, and 0.05 error tolerance	Public hospitals	To investigate critical thinking disposition among nurses working in Public Hospitals in Port-Said Governorate	Response rate: 100 %	Descriptive analysis; Reliability analysis; Inferential statistics (χ^2 tests)
15	Nguyen, 2021	Vietnam	Cross-sectional study	Clinical nurses	Convenience sampling	Calculated using the formula: n = 1.962 × p × (1–p)/0.052, where p = .46 came from the poor level of CT among nurses in the first step and 0.05 indicated the acceptable margin of error (5.0%)	Public hospitals	To measure the level of critical thinking among Vietnamese professional nurses and to identify the related factors	Response rate: 100 %	Descriptive statistics; Reliability analysis; Confirmatory factor analysis; Inferential statistics (T-tests, ANOVA, Pearson's correlation analysis)

No	Author, Year	Country	Study design	Population	Sampling method	Sample size calculation	Type of Hospital	Aim of study	Response rate	Data analysis
16	Park, 2024	Korea	Cross-sectional study	Clinical nurses	Convenience sampling	Calculated using G*Power 3.1.9.7 with a statistical power of 0.95, a significance level of 0.05, and 12 predictors based on an effect size of 0.15 and considering an expected dropout rate of 20%,	General hospital	To investigate the association between hospital ethical climate, critical thinking disposition, and nursing task performance, and confirm the mediating effect of critical thinking disposition on these relationships	Response rate: 100%	Descriptive statistics; Reliability analysis; Inferential statistics (Pearson's correlation coefficients)
17	Polat, 2019	Turkey	Descriptive, cross-sectional study design	Nurse practitioner	Random sampling	Calculated using the type 1 error was regarded as 5%, P = 0.50, confidence level as 95%, and the margin of error as 5%	Teaching hospital	To reveal the relationships between the anxiety state of nurses, their critical thinking disposition, and decision-making strategies	Response rate: 93 %	Descriptive statistics; Reliability analysis; Exploratory and confirmatory factor analysis; Inferential statistics (Independent t-test, Mann–Whitney U test, Kruskal–Wallis one-way ANOVA,

No	Author, Year	Country	Study design	Population	Sampling method	Sample size calculation	Type of Hospital	Aim of study	Response rate	Data analysis
										Pearson's correlation test)
18	Schubert, 2012	USA	Interventional study design	Medical-surgical nurses	Cluster sampling	N/A	University medical centre	To determine whether the simulation improved nurses' knowledge of failure to rescue events and critical thinking	Response rate: 21 %	Descriptive statistics; Inferential statistics (Independent samples t tests, Mann-Whitney U test)
19	Sun, 2022	China	Cross-sectional study design	Registered nurse	Convenience sampling	N/A	Medical centre	To describe CT dispositions among newly graduated nurses with different educational background and to explore related factors	Response rate: 90.4%	Descriptive statistics; Reliability analysis; Exploratory and confirmatory factor analysis; Inferential statistics (A one-way ANOVA,

No	Author, Year	Country	Study design	Population	Sampling method	Sample size calculation	Type of Hospital	Aim of study	Response rate	Data analysis
										Spearman and Pearson's correlation)
20	Urhan, 2021	Turkey	Descriptive, cross-sectional study design	Clinical nurses	Purposive sampling	N/A	Public hospitals	To investigate clinical nurses' critical thinking levels in public hospitals and related factors	Response rate: 100 %	Descriptive statistics; Reliability analysis; Inferential statistics (t--test, ANOVA, Pearson correlation analysis, Multiple linear regression analysis)
21	Wangen-steen, 2010	Norway	Descriptive, cross-sectional study design	Registered nurse	Cluster sampling	Calculated using a power analysis with statistical power of 79.6% and statistical significance (alpha) set at 0.050 (two-tailed)	University colleges	To describe critical thinking dispositions among newly graduated nurses in Norway, and to study whether background data had any impact on critical thinking dispositions	Response rate: 33 %	Descriptive statistics; Reliability analysis; Inferential statistics (Pearson's chi-square tests, Mann–Whitney U-test)

No	Author, Year	Country	Study design	Population	Sampling method	Sample size calculation	Type of Hospital	Aim of study	Response rate	Data analysis
22	Wangen-steen, 2011	Norway	Cross-sectional study design	Registered nurse	Cluster sampling	Calculated using a power analysis with statistical power of 80% and statistical significance (alpha) set at 0.05 (two-tailed)	University colleges	To describe research utilisation among newly graduated nurses and to explore critical thinking dispositions and other individual and contextual factors	Response rate: 33 %	Descriptive statistics; Reliability analysis; Inferential statistics (Simple and multiple linear regression analysis)
23	Zuriguel-Pérez, 2019	Spain	Descriptive cross-sectional and correlational study	Clinical nurses	Stratified random sampling	N/A	Tertiary care hospital	To identify the level of critical thinking among nurses in clinical practice according to sociodemographic and professional variables	Response rate: 96.8 %	Descriptive statistics; Reliability analysis; Confirmatory factor analysis; Inferential statistics (Mann-Whitney U test, Kruskal-Wallis H test, Pearson's correlation coefficients)

No	Author, Year	Country	Study design	Population	Sampling method	Sample size calculation	Type of Hospital	Aim of study	Response rate	Data analysis
24	Zuriguel-Pérez, 2018	Spain	Cross-sectional study	Nurse managers and registered nurses	Stratified random sampling	N/A	Tertiary care hospital	To analyse the levels of critical thinking among nurse managers and registered nurses and to explore the association between these levels and socio-demographic and occupational factors	Response rate: 98.3 %	Descriptive statistics; Reliability analysis; Confirmatory factor analysis; Inferential statistics (Multivariate logistic regression)
Clinical Decision-making										
1	Abu Arra, 2023	Palestine	Cross-sectional study	Emergency nurses	Cluster sampling	Calculated using the Raosoft Sample Size Calculator, with confidence level of 95% and a 5% error margin	Government hospital	To assess the factors influencing nurses' clinical decision-making in the emergency department of Palestinian hospitals.	Response rate: 91.5%	Descriptive statistics; Reliability analysis; Inferential statistics: Multiple linear regression

No	Author, Year	Country	Study design	Population	Sampling method	Sample size calculation	Type of Hospital	Aim of study	Response rate	Data analysis
2	Al-shomrani, 2024	Saudi Arabia	Cross-sectional study	Registered nurses	Convenience sampling	Calculated using the Raosoft Sample Size Calculator	Government hospital	To investigate the relationship between psychological empowerment (PE) and CDM among staff nurses, assess staff nurses' level of PE, and examine the level of CDM among staff nurses.	Response rate: 100%	Descriptive statistics; Reliability analysis; Inferential statistics (Independent t-test, ANOVA, chi- square correlation test)
3	Arash, 2024	Iran	Cross-sectional study	Intensive care unit nurses	Stratified and convenience sampling	Calculated using the G Power software and taking into account the 20% attrition	Government hospital, Private hospital	To investigate the relationship between clinical decision-making and moral distress in nurses.	Response rate: 100 %	Descriptive statistics; Reliability analysis; Inferential statistics (Independent t-test, ANOVA)

No	Author, Year	Country	Study design	Population	Sampling method	Sample size calculation	Type of Hospital	Aim of study	Response rate	Data analysis
4	Arzani, 2016	Iran	Cross-sectional study	Perioperative nurses	Purposive sampling	N/A	University hospitals	To investigate nurses' experience and their clinical decision-making skill in operating theatre based on Benner's novice to expert model	Response rate: 100 %	Descriptive statistics; Reliability analysis; Inferential statistics (Chi-square, Pearson product-moment correlation coefficient tests)
5	Batran, 2022	Palestine	Cross-sectional study	Clinical nurses	Cluster random sampling	Calculated using the G*Power 3.1 software in which effect size = 0.11, power = 0.90, and α =0.05	Government hospital	To examine the relationship between nursing informatics competencies and CDM by taking into account nurses' individual characteristics and job-related characteristics	Response rate: 86.6%	Descriptive statistics; Reliability analysis; Inferential statistics (Multiple linear regression)

No	Author, Year	Country	Study design	Population	Sampling method	Sample size calculation	Type of Hospital	Aim of study	Response rate	Data analysis
6	Bjørk, 2011	Norway	Cross-sectional study	Clinical nurses	Convenience sampling	N/A	Educational hospital, regional hospital, Local hospital	To analyse the nurses' perceptions of clinical decision making in their clinical practice and compare differences in decision making related to nurse demographic and contextual variables.	Response rate: 45.5 %	Descriptive statistics; Reliability analysis; Inferential statistics (t-tests, Pearson's r, ANOVA, Linear regression analysis)
7	Farčić, 2020	Croatia	Cross-sectional study	Clinical nurses	Convenience sampling	N/A	University Hospital	To examine the relationship between personality traits of the core self-evaluation and clinical decision-making in nurses' profession	Response rate: 59 %	Descriptive statistics; Reliability analysis; Inferential statistics (Pearson's correlation coefficient, Hierarchical regression analysis)

No	Author, Year	Country	Study design	Population	Sampling method	Sample size calculation	Type of Hospital	Aim of study	Response rate	Data analysis
8	Junghwa Yun, 2024	Korea	Cross-sectional study	Clinical nurses	Convenience sampling	Calculated using the G*Power 3.1.9.4 in which α =0.05, power (1-β) = 0.80, multiple regression median efect size = 0.15	General hospitals, private centers	To examine the relationships among nursing professionalism, empathy, and clinical decision-making ability and the factors infuencing shared decision-making awareness	Response rate: 100 %	Descriptive statistics; Reliability analysis, Inferential statistics (independent t-test, ANOVA, Pearson's correlation coefficient, Multiple linear regression)
9	Nageshwar, 2016	India	Cross-sectional study	Clinical nurses	Purposive sampling	N/A	Tertiary care hospital	To find out perceived factors affecting clinical decision-making skills regarding post-operative pain management among staff nurses	Response rate: 100 %	Descriptive statistics; Reliability analysis.

No	Author, Year	Country	Study design	Population	Sampling method	Sample size calculation	Type of Hospital	Aim of study	Response rate	Data analysis
10	Rababah, 2024	Jordan	Cross-sectional design	Registered nurses	Purposive sampling	Calculated using the G*Power with alpha = 0.05, power = 0.80, medium efect size = 0.15	General hospital	To investigate the potential predictors of clinical decision-making in registered nurses	Response rate: 69.5%	Descriptives statistics; Reliability analysis; Inferential statistics (Pearson's r coefcient, multiple linear regression)
11	Savci, 2021	Turkey	Descriptive and correlational study	Clinical nurses	Purposive sampling	N/A	Pandemic hospital	To evaluate the anxiety and clinical decision-making skills of nurses providing care to COVID-19 diagnosed patients during the pandemic period and the correlation between them	Response rate: 100%	Descriptive statistics; Reliability analysis; Inferential statistics (Pearson's correlation coefficient)

No	Author, Year	Country	Study design	Population	Sampling method	Sample size calculation	Type of Hospital	Aim of study	Response rate	Data analysis
12	Wu, 2016	China	Cross-sectional study	Clinical nurses	Convenience sampling	N/A	University hospitals	To investigate the influencing factors on nurses' clinical decision-making skills	Response rate: 87.14%	Descriptive statistics; Reliability analysis; Inferential statistics (Quantile regression method, Regression coefficients, OLS regression)
Critical Thinking and Clinical Decision-making										
1	Chen, 2016	Taiwan	Cross-sectional study	Nurse practitioners	Purposive sampling	N/A	Medical centre, Local hospitals	To investigate NPs' critical thinking dispositions, knowledge readiness, clinical decision-making score distribution and the factors that affect their decision-making	Response rate: 94 %	Descriptive statistics; Reliability analysis; Inferential statistics (Chi-square test or ANOVA, Pearson's correlations, Multiple hierarchical regression analysis)

No	Author, Year	Country	Study design	Population	Sampling method	Sample size calculation	Type of Hospital	Aim of study	Response rate	Data analysis
2	Dewi, 2021	Indonesia	Cross-sectional, descriptive correlational study	Clinical nurses	Simple random sampling	Calculated using the Slovin formula	General Hospital	To identify the relationship between nurses critical thinking and clinical decision-making abilities with the quality of nursing han-dover	Response rate: 100 %	Descriptive statistics; Reliability analysis; Inferential statistics (Pearson's correlation test)
3	Ludin, 2018	Malaysia	Cross-sectional study	Critical care nurses, emergency nurses	Purposive sampling	Calculated using the Raosoft Sample Size Calculator, with confidence level of 95% and a 5% error margin	Tertiary hospital	To understand whether critical care nurses' critical thinking disposition affects their clinical decision-making skills	Response rate: 94.9%	Descriptive statistics; Reliability analysis; Inferential statistics (One-way ANOVA test, Pearson's correlation analysis)

No	Author, Year	Country	Study design	Population	Sampling method	Sample size calculation	Type of Hospital	Aim of study	Response rate	Data analysis
4	Rababa, 2021	Jordan	Descriptive correlational study	Critical care nurses, emergency nurses	Convenience sampling	Calculated using G*Power analysis, with the anticipated effect size set at 0.25, a at 0.05 and statistical power at 0.8	University hospital	To examine nurses' critical thinking and decision-making skills related to pain management and their association with nurses' characteristics	Response rate: 100%	Descriptive statistics; Reliability analysis; Inferential statistics (Independent t-test, One-way ANOVA)
CT: Critical thinking; CDM: Clinical decision-making; ICU: Intensive care unit; N/A: Not available; ANOVA: Analysis of variance; RN: Registered nurse; α: Statistical significance (alpha); OLS: Ordinary Least Squares regression.										

Table S5: The finding score and predictive factors

No	Author, Year	Aim of study	Findings			
			Results of the observed skills		Predictive factors influenced the study	
			Level	Score	Individual	Group

Critical thinking skill						
1	Ali-Abadi, 2020	To compare the level of CT in medical- surgical and ICU nurses and investigate the factors explaining it	Poor	Med Surg: 9.12 ± 2.99; ICU: 8.68 ± 2.84	Gender	N/A
2.	Ates, 2023	To investigate the relationship between nurses' CT skills and job performance	Moderate	337.27 ± 39.96	Age, Experience	N/A
3	Bordbar, 2024	To investigate the state of critical thinking and its effect on moral courage and moral sensitivity in nurses	Weak	188.16 ± 8.22	Age, Gender, Employment status	N/A
4	Chang, 2011	To examine the relationships between CT ability and nursing competence	Middle	40.16 ± 5.95	Age, Education, Experience	N/A
5	Chen, 2019	To examine whether professional qualifications would affect self-reflection and CT in the experienced registered nurses	N/A	N/A	Age, Experience	N/A

6	Chen, 2020	To explore the relationship between CT disposition and research competence among clinical nurses	Positive general CTD	291.5 ± 29.2	N/A	N/A
7	Feng, 2010	To measure the CT competence, CT disposition and to explore the related factors	Tw-WGCT: N/A; TCTDI: Partially developed	Tw-WGCT: The cumulative average score was 61.8; TCTDI: N/A	Age, Experience, Nurses clinical ladder	N/A
8	Futami, 2020	To explore the organizational and personal factors related to CT disposition	N/A	3.3 ± 0.4	Age, experience, certification, education, seminar	Emergency unit

9	Hicks, 2003	To examine the relationship among the concepts of clinical knowledge, CT dispositions and skills, and decision-making consistency across decision tasks of varying complexity	CCTDI: N/A; CCTST: High but was drop from further analyses due to low internal consistency reliability; DAQ: A significant decrease in decision-making consistency between the low- and high-complexity tasks was found	CCTDI: 295 ± 19.9; CCTST: 17.2 ± 3.5; DAQ: 31% demonstrating consistency in the low-complexity task and 11% demonstrating consistency in the high-complexity task	N/A	N/A
10	Hsu, 2017	To explore the CTD of nurse practitioners and related factors	N/A	4.7	Age, experience, education, knowledge readiness, on-the-job training	N/A

11	Kaya, 2012	To determine the CT dispositions of emergency nurses and any influencing factors related to CT	Medium	246.76 ± 23.24	Gender, education	N/A
12	Lang, 2013	To examine CT skills among registered nurses who work in a military hospital	Satisfactory	21.69 ± 4.63	Race	N/A
13	Lee, 2022	To explore the effectiveness of a digital learning management system in enhancing intensive care unit nurses' critical care knowledge and CT tendency	N/A	4.86 ± 0.64	Continuous professional training, Education	N/A
14	Mahmoud, 2017	To investigate CT disposition among nurses working in Public Hospitals in Port-Said Governorate	Average	257.05 ± 20.16	N/A	N/A
15	Nguyen, 2021	To measure the level of CT among Vietnamese professional nurses and to identify the related factors	Moderate	333.86 ± 40.22	Age, race, education, duration of working, gender, position	N/A

16	Park, 2024	To investigate the association between hospital ethical climate, critical thinking disposition, and nursing task performance, and confirm the mediating effect of critical thinking disposition on these relationships	N/A	97.74 ± 10.70	N/A	N/A
17	Polat, 2019	To reveal the relationships between the anxiety state of nurses, their CT disposition, and decision-making strategies	Low	201.74 ± 23.890	N/A	N/A
18	Schubert, 2012	To determine whether the simulation improved nurses' knowledge of failure to rescue events and CT	N/A	N/A	N/A	N/A
19	Sun, 2022	To describe CT dispositions among newly graduated nurses with different educational background and to explore related factors	Negative disposition	N/A	Age, education	N/A
20	Urhan, 2021	To investigate clinical nurses' CT levels in public hospitals and related factors	Moderate	358.78 ± 38.10	Education, daytime shift, position	Educational hospital type

21	Wangensteen, 2010	To describe critical thinking dispositions among newly graduated nurses in Norway, and to study whether background data had any impact on CT dispositions	High (Positive inclination towards CT)	300.3 ± 24.78	Age, education	Community health care
22	Wangensteen, 2011	To describe research utilisation among newly graduated nurses and to explore CT dispositions and other individual and contextual factors	N/A	N/A	N/A	N/A
23	Zuriguel-Pérez, 2019	To identify the level of CT among nurses in clinical practice according to sociodemographic and professional variables	Moderate	362 ± 33.4	Age, shift works, experience, education	Critical care unit

24	Zuriguel-Pérez, 2018	To analyse the levels of CT among nurse managers and registered nurses and to explore the association between these levels and socio-demographic and occupational factors	Moderate	Md = 363 [IQR = 340–386]	Age, education, work shift	N/A
Clinical Decision-making skills						
1	Abu Arra, 2023	To assess the factors influencing nurses' clinical decision-making in the emergency department of Palestinian hospitals.	High	3.3 ± 0.23	Education, working hours	N/A
2	Al-shomrani, 2024	To investigate the relationship between psychological empowerment (PE) and CDM among staff nurses, assess staff nurses' level of PE, and examine the level of CDM among staff nurses.	N/A Flexible decision-making capability	70.79 ± 3.56	Age, education, years of experience	N/A

3	Arash, 2024	To investigate the relationship between clinical decision-making and moral distress in nurses.	N/A Systematic analytical decision-making capability	60.98 ± 10.25	Education, contractual employment status	N/A
4	Arzani, 2016	To investigate nurses' experience and their clinical decision-making skill in operating theatre based on Benner's novice to expert model	N/A	102.3 ± 15.3	Age, Experience	N/A
5	Batran, 2022	To examine the relationship between nursing informatics competencies and CDM by taking into account nurses' individual characteristics and job-related characteristics	Low	2.59 ± 0.38	N/A	N/A

6	Bjørk, 2011	To analyse the nurses' perceptions of clinical decision making in their clinical practice and compare differences in decision making related to nurse demographic and contextual variables.	N/A Quasi-rational decision-making style	70.65 ± 4.35	Experience, Education, Age, Gender	Surgical unit
7	Farčić, 2020	To examine the relationship between personality traits of the core self-evaluation and clinical decision-making in nurses' profession	N/A	135.8 ± 27.6	Self-esteem, Self-efficacy, Locus of control	N/A
8	Junghwa Yun, 2024	To examine the relationships among nursing professionalism, empathy, and clinical decision-making ability and the factors infuencing shared decision-making awareness	Low	138.26 ± 8.67	N/A	N/A

9	Nageshwar, 2016	To find out perceived factors affecting clinical decision-making skills regarding post-operative pain management among staff nurses	N/A	N/A	General self-esteem, Education regarding decision-making, Situation, Clinical nursing experience	N/A
10	Rababah, 2024	To investigate the potential predictors of clinical decision-making in registered nurses.	N/A Analytical–intuitive decision-making style	75.87 ± 7.19	Age, gender, percent grade, experience	N/A

11	Savci, 2021	To evaluate the anxiety and clinical decision-making skills of nurses providing care to COVID-19 diagnosed patients during the pandemic period and the correlation between them	N/A	142.22 ± 14.57	Age, Educational level, Experience	N/A
12	Wu, 2016	To investigate the influencing factors on nurses' clinical decision-making skills	Good	169.63 ± 10.79	Educational level, Experience, Total structural empowerment	N/A
Critical thinking skill and Clinical Decision-making skills						
1	Chen, 2016	To investigate NPs' critical think-<br>ing dispositions, knowledge readiness, clinical decision-making score distribution and the factors that affect their decision-making	Intuitive-analytical types	CCTDI: 73.99 ± 8.46.<br>CDMI: 165.11 ± 5.24	CDM: Knowledge readiness, Experience	CDM: Unit

2	Dewi, 2021	To identify the relationship between nurses CT and CDM abilities with the quality of nursing handover	CTQ: Moderate; CDMQ: Good	CTQ: 99.90 ± 9.038. CDMQ: 74.01 ± 3.542	N/A	N/A
3	Ludin, 2018	To understand whether critical care nurses' CT disposition affects their CDM skills	High CT High CDM	CT: 48.55 ± 8.64; CDM: 119.77 ± 13.47	CT: Age, Gender, Ethnicity, Education, Experience; CDM: Age, Experience	N/A
4	Rababa, 2021	To examine nurses' CT and decision-making skills related to pain management and their association with nurses' characteristics	CT: Low; CDM: Intuitive decision-making skills	CT: 307.3 ± 50.9; CDM:78.9 ± 9.9	CT: Experience, Education; CDM: Experience, Education, CT level	N/A

CT: Critical thinking; CDM: Clinical decision-making; Med: Medical; Surg: Surgical; ICU: Intensive care unit; N/A: Not available; CTD: Critical thinking disposition; Tw-WGCT: Taiwan Watson -Glaser Critical Thinking Appraisal; TCTDI: Taiwan Critical Thinking Disposition Inventory; CCTDI: California Critical Thinking Disposition Inventory; CCTST: California Critical Thinking Skills Test; DAQ: Decision Analytic Questionnaire; Md: Median ; IQR: Interquartile range; CDMI: Clinical Decision-Making Model Inventory; CTQ: Critical Thinking Questionnaire; CDMQ: Clinical Decision-making Questionnaire.

Table S6: The measurement tools used in selected studies.

No	Author, Year	Measurement tool used							
		Instrument	Domains	Description of tool	Aim of tool	Scoring	Reliability	EFA	CFA
Critical thinking skill									
1	Ali-Abadi, 2020	The California Critical Thinking Skills Test Form-B (CCTST-FB)	Analysis, Evaluation, Inference, Inductive reasoning and Deductive reasoning	Consists of 34 multiple-choice items with one correct answer	Level of CT	< 15.8 = Poor<br>> 15.8 = High	The Cronbach's alpha coefficient: 0.87	N/A	N/A
2	Ates, 2023	Turkish version of The Nursing Critical Thinking in Clinical Practice (CTSiCPfN)	Personal, Intellectual and cognitive, Interpersonal and self management, Technical dimensions.	Consists of 109 items rated on a 4-point Likert scale, ranging from 1 (never or almost never) to 4 (always or almost always)	CT skill	Lowest = 109, Middle = 327, Highest = 436	The Cronbach's alpha: 0.98	N/A	N/A

3	Bordbar, 2024	The California Critical Thinking Disposition Inventory (CCTDI)	Truth-seeking, Inquisitiveness, Analyticity, Systematicity, Self-confidence, Cognitive maturity, Open-mindedness	Consists of 75 questions rated on a 6-point Likert scale, ranging from 1 (complete disagree) to 6 (completely agree)	CT disposition	The scores range from 75 to 450, with 75 - 200 = poor, 201 - 325 = moderate, 326 - 450 = good	The reliability coefficient: 0.62	N/A	N/A
4	Chang, 2011	The Chinese Watson-Glaser Critical Thinking Appraisal (WGCTA-CV)	Inference, Premise identification, Deduction, Interpretation, Argument evaluation	Consists of 60 items rated on a dichotomous choice (yes or no), except for the domain of 'inference', which is rated on a multiple choice with five answers	Ability of CT	The scores range from 0–60, with higher scores saying a better ability of critical thinking	The internal consistency: 0.71	N/A	N/A
5	Chen, 2019	The Yeh Taiwan Critical Thinking Disposition Inventory (TCTDI)	Analyticity, Open-mindedness, Inquisitiveness, Reflective thinking	Consists of 20 items rated on a 6-point Likert scale, ranging from 1 (less matched) to 6 (highly matched)	CT disposition	N/A	The Cronbach's alpha: 0.96	N/A	N/A

6	Chen, 2020	The Chinese Version of Critical Thinking Disposition Inventory (CTDI-CV)	Truth Seeking, Open-mindedness, Analyticity, Systematicity, Critical Thinking Self-confidence, Inquisitiveness, Cognitive Maturity	Consists of 70 items rated on a 6-point Likert scale, from 1 (completely agree) – 6 (completely disagree)	CT disposition	> 280 = positive CTD	N/A	N/A	N/A
7	Feng, 2010	The Taiwan Watson - Glaser Critical Thinking Appraisal (Tw-WGCT)<br><br>The Yeh Taiwan Critical Thinking Disposition Inventory (TCTDI)	Tw-WGCT: Inference, Recognition of assumptions, Deduction, Interpretation, and Evaluation of arguments<br><br>TCTDI: Inquisitiveness, System activity analyticity, Open-mindedness, Reflective thinking	Tw-WGCT: Consists of 40 items with each answer worth 2.5 points each<br><br>TCTDI: Consists of 10 items rated on a 5-point Likert scale, from 1 (unable to accomplish) - 5 (completely accomplished)	CT competence<br><br>CT disposition	Tw-WGCT: The total score was 100 points.<br><br>TCTDI: N/A	Tw-WGCT: The Cronbach's alpha coefficient: ranging from 0.84 to 0.90.<br><br>TCTDI: The Cronbach's alpha coefficient: 0.85	N/A	N/A
8	Futami, 2020	The Japanese Critical Thinking Disposition Scale (JCTDS)	Awareness of logical thinking, Inquirymindedness, Objectiveness, Evidence-based judgment	Consists of 33 items rated on a 5-point Likert scale, ranging from 1 (disagree) to 5 (agree)	CT disposition	The total score is the average of the subscales. A higher score indicates a greater tendency for CT	The Cronbach's alpha coefficient: 0 .90	N/A	N/A

9	Hicks, 2003	California Critical Thinking Disposition Inventory (CCTDI)	CCTDI: Open-mindedness, Self-confidence, Maturity, Analyticity, Systematicity, Inquiry, Truth seeking	CCTDI: Consists of 75 items rated on a 6-point Likert scale	CCTDI: CT disposition.	CCTDI: <280 indicates an overall disposition away from CT, > 350 indicates an overall consistent disposition toward CT	CCTDI: 0.84.	N/A	N/A
		California Critical Thinking Skills Test (CCTST)	CCTST: Inference, Evaluation, Analysis,	CCTST: Consists of 34 items with 1 correct response per question	CCTST: CT skill	CCTST: Total score ranges from 0 to 34 point	CCTST: 0.47 (KR-20)	N/A	N/A
		Decision Analytic Questionnaire (DAQ)	DAQ: Intuitive decision processes, Analytical decision processes	DAQ: Consists of 2 clinical scenarios with 57 items	DAQ: CDM consistency	DAQ: N/A	DAQ: N/A	N/A	N/A
10	Hsu, 2017	The Yeh Critical Thinking Disposition (CTD)	Systematicity and analyticity, Openness and empathy, Intellectual inquisitiveness, Holistic and reflective thinking	Consists of 20 items rated on a 6-point Likert scale, from 1 (never) to 6 (always)	CT disposition	The total score is the average of the subscales.	The Cronbach's alpha coefficient: 0 .92	N/A	N/A

11	Kaya, 2012	The California Critical Thinking Disposition Inventory (CCTDI)	Analyticity, Open-mindedness, Inquisitiveness, Self-confidence, Truth-seeking, Systematicity	Consists of 51 items rated on 6-point Likert scale, ranging from 1 (strongly disagree) to 6 (strongly agree)	CT disposition	< 240 = Low level, 240-300 = Medium level, > 300 = High level	The total Cronbach's alpha coefficient: 0.81	N/A	N/A
12	Lang, 2013	The Health Sciences Reasoning Test (HSRT)	Inductive reasoning, Deductive reasoning, Inferential reasoning, Analytical ability, Evaluation	Consists of 33 items with each answer worth 1 point each	Level of CT skills	Total score can range from 0 to 33; 0 - 5 = Poor, 16 - 24 = satisfactory, 25 or above = strong CT	N/A	N/A	N/A
13	Lee, 2022	The critical thinking questionnaire (CTQ)	N/A	Consists of 6 items rated on a 5-point Likert scale, ranging from 1 (strongly disagree) to 5 (strongly agree)	CT ability	N/A	The Cronbach’s alpha value: 0.71.	N/A	N/A
14	Mahmoud, 2017	The California Critical Thinking Disposition Inventory (CCTDI)	Truth seeking, Open mindedness, Analyticity, Systematicity, Self-confidence, Inquisitiveness, Maturity	Consists of 75 items rated on a 6-point Likert scale, ranging from 1 (strongly disagree) to 6 (strongly agree)	CT disposition	< 210 = negative disposition; 210 - 279 = ambivalence toward disposition; 280 - 420 = positive disposition	The Cronbach Alpha value: 0.78	N/A	N/A

15	Nguyen, 2021	The Nursing Critical Thinking in Clinical Practice (N-CT-4 Practice)	Personal, Intellectual and Cognitive, Interpersonal and Self-management, Technical Dimensions.	Consists of 109 items rated on a 4-point Likert scale, ranging from 1 (never or almost never) to 4 (always or almost always)	CT ability	< 329 = Low, 329 – 395 = moderate, > 395 = High	The Cronbach's alpha value: 0.98	N/A	χ2/df= 2.87, RMSEA= 0.059, SRMR = 0.063, CFI = 0.73, TLI = 0.72
16	Park, 2024	The Critical Thinking Disposition Scale (CTDS)	Intellectual eagerness/curiosity, Prudence, Self-confidence, Systematicity, Intellectual fairness, Healthy skepticism, Objectivity	Consists of 27 items rated on a 5-point Likert scale, ranging from 1 (I do not agree at all) to 5 (Absolutely agree)	CT disposition	Total score ranging from 27 to 135	The Cronbach's alpha value: 0.90.	N/A	N/A
17	Polat, 2019	The Turkish California Critical Thinking Disposition Inventory (Turkish CCTDI)	Systematicity, Open-mindedness, Analytics, Seeking Truth, Self–confidence, Inquisitiveness	Consists of 51 items rated on 6-point Likert scale, between 1 and 6	CT disposition	< 240 = Low, > 300 = High	The Cronbach's alpha value: 0.88	KMO value: 0.43, percentage of variance explained: 39%, The factor load value: between 0.33 - 0.5	ChiSq/df = 5.5, AGFI = 0.71, Root mean square residual value = 0.113

18	Schubert, 2012	The Learning Transfer Tool (LTT)	N/A	Consists of 13 items that require nurses to self-assess their ability to plan and prioritize care, evaluate and communicate those priorities, and recognize and interpret deviations	CT skill	N/A	N/A	N/A	N/A
19	Sun, 2022	The Chinese Version of Critical Thinking Dispositions Inventory (CTDI-CV)	Open-mindedness, Analyticity, Cognition, Maturity, Truthseeking, Systematicity, Inquisitivenes and self-confidence	Consists of 70 items rated on a 6-point Likert scale, from 1 (completely agree) – 6 (completely disagree)	CT disposition	> 350 indicate strong disposition, 280 - 350 positive inclinations, 210–279 indicate ambivalent, < 210 strong opposition	The Cronbach's alpha value: 0.938	N/A	N/A
20	Urhan, 2021	The Turkish Version of Nursing Critical Thinking in Clinical Practice (N-CT-4 Practice Tv)	Personal Characteristics, Intellectual and Cognitive Abilities, Interpersonal Abilities and Self-management, Technical Abilities.	Consists of 109 items rated on a 4-point Likert scale, ranging from 1 (strongly disagree) to 4 (strongly agree)	CT skill	Lowest = 109, Middle = 327, Highest = 436	The internal consistency coefficient value: 0.978	N/A	N/A

21	Wangensteen, 2010	The California Critical Thinking Disposition Inventory (CCTDI)	Truth-seeking, Open-mindedness, Analyticity, Systematicity, Self-confidence, Inquisitiveness, Maturity	Consists of 75 items rated on a 6-point Likert scale, ranging from 1 (strongly agree) to 6 (strongly disagree)	CT disposition	< 210 = Strong opposition towards CT, 210 - 279 = ambivalent, 280 - 350 = positive inclination, > 350 = strong disposition	The Cronbach's alpha value: 0.83	N/A	N/A
22	Wangensteen, 2011	The California Critical Thinking Disposition Inventory (CCTDI)	Truth-seeking, Open-mindedness, Analyticity, Systematicity, Self-confidence, Inquisitiveness, Maturity	Consists of 75 items rated on a 6-point Likert scale, ranging from 1 (strongly agree) to 6 (strongly disagree)	CT disposition	< 210 = Strong opposition towards CT, 210 - 279 = ambivalent, 280 - 350 = positive inclination, > 350 = strong disposition	The Cronbach's alpha value: 0.83	N/A	N/A
23	Zuriguel-Pérez, 2019	The Nursing Critical Thinking in Clinical Practice (N-CT-4 Practice)	Personal characteristics, Intellectual and cognitive abilities, Interpersonal abilities and self-management, Technical abilities.	Consists of 109 items rated on a 4-point Likert scale, ranging from 1 (never or almost never) to 4 (always or almost always)	Level of CT	< 328.6 = Low, 328.7 - 395.3 = Moderate, > 395.4 = High level	The Cronbach's alpha value: 0.96	N/A	RMSEA = 0.055, SRMR= 0.65, $\chi 2/df$ = 1.95, CFI = 0.629, TLI = 0.621

24	Zuriguel-Pérez, 2018	The Nursing Critical Thinking in Clinical Practice (N-CT-4 Practice)	Personal characteristics, Intellectual and cognitive abilities, Interpersonal abilities and self-management, Technical abilities.	Consists of 109 items rated on a 4-point Likert scale, ranging from 1 (never or almost never) to 4 (always or almost always)	Level of CT	< 328.6 = Low, 328.7 - 395.3 = Moderate, > 395.4 = High level	The Cronbach's alpha value: 0.96	N/A	RMSEA = 0.055, SRMR= 0.65, χ2/df = 1.95, CFI = 0.629, TLI = 0.621
Clinical Decision-making skills									
1	Abu Arra, 2023	The Clinical Decision Making in Nursing Scale (CDMNS)	Search for alternatives or options, Canvassing of objectives and values, Evaluation and reevaluation of consequences, Search for information and unbiased assimilation of new information	Consists of 40 items rated on a 5-point Likert scale, including "always", "frequently", "occasionally", "seldom", and "never".	Level of CDM	A mean score of ≥3.00 indicates high clinical decision-making, and a mean score of <3.00 indicates low decision-making	The Cronbach's alpha value: 0.89	N/A	N/A
2	Al-shomrani, 2024	The 24-item Nursing Decision Making Instrument	N/A	Consists of 24 items rated on a 5-point Likert scale, including "almost always", "often", "sometimes", "rarely", and "almost never".	Decision-making style	>78 indicate intuitive decision-making capability; between 68 and 78 indicate flexible decision-making capability;	The Cronbach's alpha value: 0.89	N/A	N/A

						and <67 indicate analytically oriented decision-making capability.			
3	Arash, 2024	The 24-item Nursing Decision Making Instrument	N/A	Consists of 24 items rated on a 5-point Likert scale, including “always”, "often", "average", "rarely", and "never".	Decision-making style	24– 67 indicate systematic analytical decision-making, 68 and 77 indicate intuitive analysis, 78–120 indicate interpretive intuition	The Cronbach's alpha value: 0.89	N/A	N/A
4	Arzani, 2016	The Clinical Decision Making in Nursing Scale (CDMNS)	Search for alternatives or options, Canvassing of objectives and values, Evaluation and re-evaluation of consequences, Search for information and unbiased assimilation of new information	Consists of 40 items rated on a 5-point Likert scale, including “always”, “usually”, “sometimes”, “hardly ever”, and “never”	Level of CDM	The highest and lowest possible scores are 40 and 200, there is no cut-off point	The Cronbach's alpha value: 0.82	N/A	N/A

5	Batran, 2022	The Clinical Decision Making in Nursing Scale (CDMNS)	Search for alternatives or options, Canvassing of objectives and values, Evaluation and re-evaluation of consequences, Search for information and unbiased assimilation of new information	Consists of 40 items rated on a 5-point Likert scale, including "always", "frequently", "occasionally", "seldom", and "never"	Level of CDM	A mean score of ≥ 3.00 indicates high clinical decision-making, and a mean score of <3.00 indicates low decision-making	The Cronbach's alpha value: 0.89	N/A	N/A
6	Bjørk, 2011	The 24-item Nursing Decision Making Instrument	Collection of data, Data processing and identification of problems, Plan of action and implementation of the plan, Monitoring, and evaluation	Consists of 24 items rated on a 5-point Likert scale, ranging from 1 (almost never) to 5 (almost always)	Decision-making style	24–67 indicate analytical-systematic decision-making, 68–77 indicate quasi-rational decision-making, and 78–120 say intuitive-interpretive decision-making	The Cronbach's alpha value: 0.863	N/A	N/A

7	Farčić, 2020	The Clinical Decision-Making Nurses Scale (CDMNS)	Search for alternatives or options, Canvassing of objectives and values, Evaluation and re-evaluation of consequences, Search for information and unbiased assimilation of new information	Consists of 40 items rated on a 5-point Likert scale, ranging from 1 (never) to 5 (always)	Level of CDM	The total score ranged from 40 to 200, there is no cut-off point	The Cronbach's alpha value: 0.94	N/A	N/A
8	Junghwa Yun, 2024	The Clinical Decision-Making Nurses Scale (CDMNS)	Examination of alternatives and options, Review of values and goals, Examination of information, harmonization of new information, Assessment and reassessment of conclusions	Consists of 40 items rated on a 5-point Likert scale, ranging from 1 (strongly disagree) to 5 (strongly agree)	Level of CDM	The total score ranged from 40 to 200.	The Cronbach's alpha value: 0.72.	N/A	N/A

9	Nageshwar, 2016	The 36-item Questionnaire of Factors Affecting CDM	Relationship with ward in charge, Perception of decision-making, Locus of control, Clinical nursing experience, Situation, Education regarding decision-making, Cognitive processes, Stress, Role-modelling, General self-esteem	Consists of 36 items rated on a 5-point Likert scale, including "strongly agree", "agree", "undecided", "disagree" and "strongly disagree"	Factors affecting CDM	The minimum score was 36 and the maximum was 180	The internal consistency coefficient value: 0.75	N/A	N/A
10	Rababah, 2024	The 24-item nurse decision-making instrument-revised	N/A	Consists of 24 items rated on a 5-point Likert scale, ranging from 1 “almost never” to 5 “almost always”.	Decision-making style	24–67 indicate analytical–systematic, 68–77 indicate quasirational, 78–120 indicate intuitive–interpretive	The Cronbach's alpha value: 0.96.	N/A	N/A
11	Savci, 2021	The Clinical Decision Making in Nursing Scale (CDMNS)	Search for alternatives or options, Canvassing of objectives and values, Evaluation and re-evaluation of consequences, Search for information and unbiased assimilation of new information	Consists of 40 items rated on a 4 -point Likert scale. Each subscale is composed of 10 items	Level of CDM	The total score ranged from 40 to 200, there is no cut-off point	The Cronbach's alpha value: 0.82	N/A	N/A

12	Wu, 2016	The Practical Knowledge Inventory for Nurses (PKIN)	Managing oneself, Managing others, Managing tasks	A paper-and-pencil test that contains 10 scenarios rated on a 7-point Likert-type scale, ranging from 1 (extremely inadequate) to 7 (extremely adequate)	Nurses' ability levels	The total score ranges from 0 to 220. A higher score on the scale indicates a higher level of CDM skills	The Cronbach's alpha value: 0.7	N/A	N/A
Critical thinking skill and Clinical Decision-making skills									
1	Chen, 2016	The Yeh's Chinese version of the California Critical Thinking Disposition Inventory (CTDI)<br><br>The Clinical Decision-Making Model Inventory (CDMI)	CCTDI: Systematicity and analyticity, Openness and empathy, Intellectual inquisitiveness, holistic and reflective traits<br><br>CDMI: Reflect the 4 stages of the decision-making process: Data collection, Data processing and problem identification, Plan of action, Plan implementation,	CCTDI: Consists of 20 items rated on a 6-point Likert Scale, ranging from 1 (never) to 6 (always)<br><br>CDMI: Consists of 56 items rated on a 5-point Likert scale, ranging from 1 (almost never) to 5 (almost always)	CCTDI: CT disposition<br><br>CDMI: CDM abilities	CCTDI: Higher scores indicate a greater predisposition towards CT<br><br>CDMI: 56–160 indicate analytical tendencies, 161–164 indicate analytical-intuitive tendencies, 166–170 indicate	CCTDI: The Cronbach's alpha value is 0.93<br><br>CDMI: The Cronbach's alpha value ranged between 0.90 – 0.92	CCTDI: N/A<br><br>CDMI: N/A	CCTDI: N/A<br><br>CDMI: N/A

			monitoring and evaluation			intuitive-analytical tendencies, 171–280 indicate intuitive tendencies.			
2	Dewi, 2021	The Critical Thinking Questionnaire (CTQ)<br><br>The Clinical Decision-making Questionnaire (CDMQ)	CTQ: Interpretation, Analysis, Inference, Evaluation, Explanation, Self-regulation<br><br>CDMQ: Identifying the problem, creating criteria, looking for alternative solutions, conducting an	CTQ: N/A<br><br>CDMQ: N/A	CTQ: CT ability<br><br>CDMQ: CDM ability	CTQ: N/A<br><br>CDMQ: N/A	The Cronbach alpha values >0.60<br><br>The Cronbach alpha values >0.60	CTQ: N/A<br><br>CDMQ: N/A	CTQ: N/A<br><br>CDMQ: N/A

			evaluation, Choosing alternatives						

3	Ludin, 2018	The Short Form-Critical Thinking Disposition Inventory-Chinese Version (SF-CTDI-CV)	SF-CTDI-CV: Systematic analysis, Thinking within the box, Thinking outside the box	SF-CTDI-CV: Consists of 18 items rated on a 5-point Likert Scale ranging from 1 (completely disagree) to 5 (completely agree);	SF-CTDI-CV: CT disposition	≤ 35.75 indicate low levels, while >35.75 indicate high levels	SF-CTDI-CV: The Cronbach's alpha was 0.740	SF-CTDI-CV: N/A	SF-CTDI-CV: N/A
		The Clinical Decision-making Nursing Scale (CDMNS)	CDMNS: Search for alternatives or options, Search for information and unbiased assimilation of new information, Evaluation and re-evaluation of consequences, Canvassing of objectives	CDMNS: Consists of 40 items rated on a 5-point Likert Scale ranging from 1 (strongly agree) to 5 (strongly disagree)	CDMNS: CDM level	The minimum and maximum possible scores were 40 and 200 respectively. Scores < 50% indicate lower levels, while scores > 50% of the total maximum score are considered to indicate higher levels of CDM	CDMNS: The Cronbach's alpha was 0.797	CDMNS: N/A	CDMNS: N/A

4	Rababa, 2021	The Critical Thinking Self-Assessment Scale (CTSAS)	CTSAS: Inference, Self-regulation, Analysis, Evaluation, Interpretation, Explanation	CTSAS: Consists of 115 items rated on a 7-point Likert Scale ranging from 0 (never) to 6 (always)	CTSAS: CT skills	CTSAS: The total possible score ranges from 0 to 690	CTSAS: The Cronbach's alpha was 0.85	CTSAS: N/A	CTSAS: N/A
		The Nursing Decision-Making Instrument (NDMI)	NDMI: N/A	NDMI: Consists of 24 items rated on a 5-point Likert Scale ranging from 1 (never) to 5 (always)	NDMI: CDM modes	NDMI: <67 indicating analytical decision making, 68 to 78 indicating flexible analytical-intuitive decision making, and > 78 indicating intuitive decision making	NDMI: The Cronbach's alpha was 0.82	NDMI: N/A	NDMI: N/A

CT: Critical thinking; CDM: Clinical decision-making; EFA: Exploratory factor analysis; CFA: Confirmatory factor analysis; CCTST-FB: California Critical Thinking Skills Test Form-B; N-CT-4 Practice: Nursing Critical Thinking in Clinical Practice Questionnaire; WGCTA: Watson-Glaser Critical Thinking Appraisal; TCTDI: Taiwan Critical Thinking Disposition Inventory; CTDI-CV: Chinese Version of Critical Thinking Disposition Inventory; Tw-WGCT: Taiwan Watson -Glaser Critical Thinking Appraisal; JCTDS: Japanese Critical Thinking Disposition Scale; CCTDI: California Critical Thinking Disposition Inventory; CCTST: California Critical Thinking Skills Test; DAQ: Decision Analytic Questionnaire; CTDS: Critical Thinking Disposition Scale; HSRT: Health Sciences Reasoning Test; CTQ: Critical Thinking Questionnaire; LTT: Learning Transfer Tool; CTSiCPfN/N-CT-4 Practice Tv: Turkish Version of Nursing Critical Thinking in Clinical Practice Questionnaire; CDMNS: Clinical Decision Making in Nursing Scale; PKIN: Practical Knowledge Inventory for Nurses; CDMI: Clinical Decision-Making Model Inventory; CDMQ: Clinical Decision-making Questionnaire; SF-CTDI-CV: Short Form- Critical Thinking Disposition Inventory-Chinese Version; CTSAS: Critical Thinking Self-Assessment Scale; NDMI: Nursing Decision-Making Instrument; N/A: Not Available
